# Application of the lumped age-class technique to studying the dynamics of malaria-mosquito-human interactions

**DOI:** 10.1186/1475-2875-6-98

**Published:** 2007-07-30

**Authors:** Penny A Hancock, H Charles J Godfray

**Affiliations:** 1 Natural Environment Research Council Centre for Population Biology & Division of Biology, Imperial College London, Silwood Park Campus, Ascot, Berks, SL5 7PY, UK; 2 Department of Zoology, University of Oxford, South Parks Road, Oxford, OX1 3PS, UK

## Abstract

A series of models of malaria-mosquito-human interactions using the Lumped Age-Class technique of Gurney & Nisbet are developed. The models explicitly include sub-adult mosquito dynamics and assume that population regulation occurs at the larval stage. A challenge for modelling mosquito dynamics in continuous time is that the insect has discrete life-history stages (egg, larva, pupa & adult), the sub-adult stages of relatively fixed duration, which are subject to very different demographic rates. The Lumped Age-Class technique provides a natural way to treat this type of population structure. The resulting model, phrased as a system of delay-differential equations, is only slightly harder to analyse than traditional ordinary differential equations and much easier than the alternative partial differential equation approach. The Lumped Age-Class technique also allows the natural treatment of the relatively fixed time delay between the mosquito ingesting *Plasmodium *and it becoming infective. Three models are developed to illustrate the application of this approach: one including just the mosquito dynamics, the second including *Plasmodium *but no human dynamics, and the third including the interaction of the malaria pathogen and the human population (though only in a simple classical Ross-Macdonald manner). A range of epidemiological quantities used in studying malaria such as the vectorial capacity, the entomological inoculation rate and the basic reproductive number (*R*_0_) are derived, and examples given of the analysis and simulation of model dynamics. Assumptions and extensions are discussed. It is suggested that this modelling framework may be a natural and useful tool for exploring a variety of issues in malaria-vector epidemiology, especially in circumstances where a dynamic representation of mosquito recruitment is required.

## Background

The malaria pathogen can be combated either in its human host or mosquito vector and both strategies have received enormous attention over the years. The interaction between *Anopheles *and *Plasmodium *is complex and non-linear, even when the further complexities of mosquito-human interactions are omitted, and population biology models have proved important in understanding the quantitative epidemiology of the association. One strand of work has used computer simulation models to produce highly detailed descriptions of the interaction, which also normally include meteorological drivers [e.g. [1,2]]. Another strand, that dates back to the pioneering work of Ross [3] & McDonald [4], models the interaction using much simpler sets of equations that sacrifice detail for mathematical tractability and analytical insight. This second school of modelling has recently been reviewed in this journal by Smith & McKenzie [5].

The standard technique for developing relative simple mathematical descriptions of mosquito-*Plasmodium *interactions is to model the system as a set of ordinary differential equations (ODEs). This is an immensely powerful approach, and has led to many insights into the factors that affect malaria prevalence and control [4,6-10]. However, there are aspects of the life-cycle of the mosquito and *Plasmodium *that are difficult to incorporate within an ODE framework. First, the life history of the vector is divided into four stages – egg, larva, pupa and adult – with very different demographic parameters. Mortality rates are highly likely to be stage specific, especially as adult and juvenile stages occupy very different micro-environments, while only the adults reproduce. The life cycle also means that there is a time-delay between reproduction and recruitment to the adult population. Second, mosquitoes that take up the malaria pathogen (the exposed class) do not immediately become infectious; there is a time delay during which the gametocytes fuse, form oocysts, and the sporozoites mature and migrate to the salivary glands. The time lags associated with mosquito development and sporozoite maturation are not straightforward to model using ODEs.

These problems have been overcome or circumvented in a number of different ways. For mosquito development the normal practice is to ignore the juvenile stages and to assume that adult mosquitoes emerge at a constant rate [6] or at a rate that varies cyclically with the seasons [7,9,11]. It is generally thought that mosquito populations are regulated by processes operating on the juvenile stages, which might justify this assumption. However, it is not possible to analyse fully the impact of processes that affect juvenile recruitment such as larval habitat modification with this type of model.

A variety of approaches have been taken to model sporozoite maturation. In the simplest mosquitoes are divided into susceptible and infectious classes (an SI model) and the exposed class is either ignored or incorporated only implicitly as a mortality term reducing the flow of individuals from the susceptible to infectious classes [4,5]. Next an explicit exposed class can be included (giving an SEI model), but maturation out of the stage is assumed to be a linear function of the density of exposed. This introduces a time delay, but as individuals are "at risk" of maturing into the infectious stage immediately they become exposed, it only poorly replicates the relative fixed development time observed in real infections. A much better approach is to introduce multiple exposed stages through which each individual has to transit before it can become infectious [7]. The residence times in the exposed stage is then Gamma distributed and if the mean length is *T*_*E *_then the variance is TE2
 MathType@MTEF@5@5@+=feaafiart1ev1aaatCvAUfKttLearuWrP9MDH5MBPbIqV92AaeXatLxBI9gBaebbnrfifHhDYfgasaacH8akY=wiFfYdH8Gipec8Eeeu0xXdbba9frFj0=OqFfea0dXdd9vqai=hGuQ8kuc9pgc9s8qqaq=dirpe0xb9q8qiLsFr0=vr0=vr0dc8meaabaqaciaacaGaaeqabaqabeGadaaakeaacqWGubavdaqhaaWcbaGaemyraueabaGaeGOmaidaaaaa@300F@/*n *which can be made arbitrarily small by increasing the number of stages, *n*. This much more realistic representation of the delay does though come with the disadvantage of having to deal with a much larger system of ODEs, for example Smith et al. [7] used *n *= 64. A rather different approach, used as much for data analysis as for population modelling, is to discretise the problem. This may be done by writing down recurrence equations for the density of mosquitoes that have been in the exposed stage for different number of days, or the time step may not be a day but the length of the gonotrophic cycle [12-14]. Discretisation is also how sporozoite maturation is modelled in large simulation studies. Macdonald [4,6] used delay-differential equations (DDEs) to describe adult mosquito stages (a similar approach is used below) and to derive different epidemiological quantities (see also the recently-published ref [15] concerning mosquito-dengue interactions)

Variation in demographic parameters and infection status with time can both be considered problems in age-structured population dynamics where one needs to characterise a population not only by a series of single-variable quantities (i.e. the number of individuals at time *t *in class *x*) but by a series of double- or even multiple-variable quantities (i.e. the number of individuals of age *a *and/or duration of infection *b *at time *t *in class *x*). The mathematically natural way to approach such problems is to use systems of partial differential equations (PDEs) [16]. However, the analysis of non-linear PDEs is both analytically and numerically challenging, and these methods have rarely been applied in vector population biology.

The Lumped Age-Class technique is an approach that combines some of the advantages of the ODE and PDE approaches. It assumes that the life cycle of an organism is divided into stages during which its demographic parameters can be assumed to remain constant (as in the ODE models above), but it also assumes that individuals remain within developmental stages for fixed or minimum periods of time (as in PDE models). This latter assumption means that developmental lags can be incorporated in a much more natural way. There are two costs of this added realism. First, the models have to be phrased as systems of delay-differential equations (DDEs) that are slightly harder to manipulate than ODEs but substantially easier than PDEs. Second, a number of extra equations need to be written down to describe the rate of change of survival through certain developmental stages, though typically this number is quite small. The Lumped Age-Class technique was invented by Gurney & Nisbet [17-19] and initially applied to understanding age-structured interactions in insect intraspecific competition. Since then it has proved particular valuable in studying interactions between insects and their parasitoids [20-24] and pathogens [25,26]. The concentration on insect systems is no coincidence as the division of the life cycle of holometabolous species into eggs, larvae (and within this stage into instars), pupae and adults with very different demographic parameters renders them very appropriate for this approach.

In this paper a Lumped Age-Class model is developed for a mosquito population that transmits malaria. The primary aim is to develop a flexible model that can be applied to a variety of problems, and to demonstrate the utility of this approach for vector-borne diseases. Concentrating on the population dynamics of the mosquito, a more detailed description of the juvenile stages than is normal is incorporated, as well as a fixed period for sporozoite maturation. In contrast, only very simple assumptions about the dynamics of malaria in humans are made, though the Discussion explores how this might be made more realistic. In the next section a series of three models of increasing complexity are described with their assumptions listed. In the following section the classic epidemiological quantities used in studying malaria such as the vectorial capacity, the entomological inoculation rate and the basic reproductive number (*R*_0_) are derived. In the penultimate section examples of using the model to study dynamics are given and the paper finishes with a Discussion. It is stressed that the aim of this article is chiefly to introduce this modelling technique to studies of malaria epidemiology rather than to model a specific mosquito-*Plasmodium *interaction.

## Methods (Model development)

**Figure 1:**
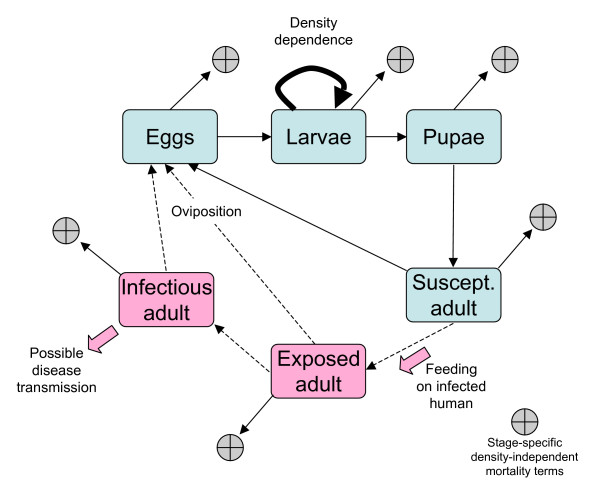
Schematic model of a stage-structured mosquito population that can transmit malaria. The mosquito life cycle is divided into egg, larval, pupal and adult female stages, the latter being subdivided into susceptible, exposed and infectious classes. In the models developed here stage-specific density-independent mortality is assumed to act on all stages, while the expected size of the mosquito population is determined by density-dependent processes operating in the larval stage. All adult classes can reproduce, though not necessarily at the same rate.

Consider the simplified mosquito life cycle in Figure 1 (some of the assumptions are relaxed later). It is assumed throughout that all population measures refer to densities of female mosquitoes unless specifically stated. The immature period is divided into eggs, larvae and pupae whose densities at time *t *are denoted by the letters *O*(*t*), *L*(*t*) and *P*(*t*) respectively (*O *for eggs or ova is used as *E *is required for exposed adults). Adult mosquitoes may be uninfected and susceptible; exposed and carrying *Plasmodium *but not yet capable of malaria transmission; or carrying the *Plasmodium *and fully infectious; these three stages will be denoted *S*(*t*), *E*(*t*) and *I*(*t*) in line with standard epidemiological terminology. It is assumed that the duration of the egg, larval, pupal and exposed adult stages are fixed and last *T*_*i *_days (*i *∈ {*O, L, P*}). All stages suffer potential different levels of density independent mortality at rates *μ*_*i*_ d^-1 ^(*i *∈ {*O, L, P, S, E, I*}) and the variables *θ*_*i *_= exp-*T*_*i*_*μ*_*I *_are introduced to denote the probability of surviving density independent mortality during stage *i*. In addition it is assumed that larvae experience density dependent mortality at a rate given by the function *g*(*L*(*t*)). Adult mosquitoes lay female eggs at a rate *λ*_*i *_d^-1 ^(*i *∈ {*S, E, I*}) that may vary with infection status; susceptible adults feed on humans at a rate *a *and pick up *Plasmodium *at a rate *c *from the fraction *x*(*t*) of humans that are infectious at time *t*. Note the simplifying assumption that adults feed and oviposit concurrently; the issue of explicitly incorporating the gonotrophic cycle is returned to in the Discussion. It is also assumed that all demographic parameters are constant within a class: thus there is the possibility of stage-specific but not age-specific adult mortality.

### Model 1, Mosquito with no Plasmodium

A basic model describing the mosquito dynamics alone (that is without the exposed and infectious adult stages) can be specified by two "balance equations" for the numbers of individuals entering and leaving the larval and adult stages and a third equation for the rate of change of survival through the larval stage.

The numbers of larval mosquito are affected by four processes: (i) they increase as eggs hatch and they decrease as (ii) individuals mature into pupae, (iii) die because of density-independent mortality, and (iv) die because of density-dependent mortality. The number of eggs hatching (i) is the product of the density of ovipositing females *T*_*O *_days ago, their per capita fecundity, and the probability of living through the egg stage (*θ*_*O*_). To calculate the numbers pupating (ii) first define the probability of living through the larval stage as *θ*_*L*_*ω*_*L*_(*t*) where the first and second terms represent density independent and dependent mortality respectively. The second is a function of time as it depends on possibly varying larval density and an exact expression for this quantity is given below. The number of larvae pupating is thus the total eggs laid *T*_*O *_+ *T*_*L *_days ago multiplied by the probability of surviving both the egg and larval stages. The numbers of larvae succumbing to density-independent mortality per unit time (iii) is *L*(*t*) *μ*_*L *_and the numbers removed by density-dependent mortality (iv) is *L*(*t*) *g*(*L*(*t*)). The balance equation for larval densities, which contains four terms corresponding to the four processes described above, is

dL(t)dt=λSS(t−TO)θO−λSS(t−TO−TL)θOθLωL(t)−μLL(t)−g(L(t))L(t).
 MathType@MTEF@5@5@+=feaafiart1ev1aaatCvAUfKttLearuWrP9MDH5MBPbIqV92AaeXatLxBI9gBaebbnrfifHhDYfgasaacH8akY=wiFfYdH8Gipec8Eeeu0xXdbba9frFj0=OqFfea0dXdd9vqai=hGuQ8kuc9pgc9s8qqaq=dirpe0xb9q8qiLsFr0=vr0=vr0dc8meaabaqaciaacaGaaeqabaqabeGadaaakeaadaWcaaqaaiabdsgaKjabdYeamjabcIcaOiabdsha0jabcMcaPaqaaiabdsgaKjabdsha0baacqGH9aqpiiGacqWF7oaBdaWgaaWcbaGaem4uamfabeaakiabdofatjabcIcaOiabdsha0jabgkHiTiabdsfaunaaBaaaleaacqWGpbWtaeqaaOGaeiykaKIae8hUde3aaSbaaSqaaiabd+eapbqabaGccqGHsislcqWF7oaBdaWgaaWcbaGaem4uamfabeaakiabdofatjabcIcaOiabdsha0jabgkHiTiabdsfaunaaBaaaleaacqWGpbWtaeqaaOGaeyOeI0Iaemivaq1aaSbaaSqaaiabdYeambqabaGccqGGPaqkcqWF4oqCdaWgaaWcbaGaem4ta8eabeaakiab=H7aXnaaBaaaleaacqWGmbataeqaaOGae8xYdC3aaSbaaSqaaiabdYeambqabaGccqGGOaakcqWG0baDcqGGPaqkcqGHsislcqWF8oqBdaWgaaWcbaGaemitaWeabeaakiabdYeamjabcIcaOiabdsha0jabcMcaPiabgkHiTiabdEgaNjabcIcaOiabdYeamjabcIcaOiabdsha0jabcMcaPiabcMcaPiabdYeamjabcIcaOiabdsha0jabcMcaPiabc6caUaaa@754A@

Now consider the numbers of adult, susceptible mosquitoes. As *Plasmodium *infection is not yet included the density of adults is affected by only two processes; maturation from the pupal stage and density independent mortality. The former equals the number of eggs laid *T*_*O *_+ *T*_*L *_+ *T*_*P *_days ago multiplied by the probability of surviving the three immature stages. The latter is simply *μ*_*s *_*S*(*t*). The balance equation is thus

dS(t)dt=λSS(t−TO−TL−TP)θOθLωL(t−TP)θP−μSS(t).
 MathType@MTEF@5@5@+=feaafiart1ev1aaatCvAUfKttLearuWrP9MDH5MBPbIqV92AaeXatLxBI9gBaebbnrfifHhDYfgasaacH8akY=wiFfYdH8Gipec8Eeeu0xXdbba9frFj0=OqFfea0dXdd9vqai=hGuQ8kuc9pgc9s8qqaq=dirpe0xb9q8qiLsFr0=vr0=vr0dc8meaabaqaciaacaGaaeqabaqabeGadaaakeaadaWcaaqaaiabdsgaKjabdofatjabcIcaOiabdsha0jabcMcaPaqaaiabdsgaKjabdsha0baacqGH9aqpiiGacqWF7oaBdaWgaaWcbaGaem4uamfabeaakiabdofatjabcIcaOiabdsha0jabgkHiTiabdsfaunaaBaaaleaacqWGpbWtaeqaaOGaeyOeI0Iaemivaq1aaSbaaSqaaiabdYeambqabaGccqGHsislcqWGubavdaWgaaWcbaGaemiuaafabeaakiabcMcaPiab=H7aXnaaBaaaleaacqWGpbWtaeqaaOGae8hUde3aaSbaaSqaaiabdYeambqabaGccqWFjpWDdaWgaaWcbaGaemitaWeabeaakiabcIcaOiabdsha0jabgkHiTiabdsfaunaaBaaaleaacqWGqbauaeqaaOGaeiykaKIae8hUde3aaSbaaSqaaiabdcfaqbqabaGccqGHsislcqWF8oqBdaWgaaWcbaGaem4uamfabeaakiabdofatjabcIcaOiabdsha0jabcMcaPiabc6caUaaa@6424@

To complete the specification of the system without *Plasmodium *an explicit representation of the probability of surviving density-dependent mortality during the larval stage is needed

ωL(t)=exp⁡(−∫t−TLtg(L(τ)) dτ).
 MathType@MTEF@5@5@+=feaafiart1ev1aaatCvAUfKttLearuWrP9MDH5MBPbIqV92AaeXatLxBI9gBaebbnrfifHhDYfgasaacH8akY=wiFfYdH8Gipec8Eeeu0xXdbba9frFj0=OqFfea0dXdd9vqai=hGuQ8kuc9pgc9s8qqaq=dirpe0xb9q8qiLsFr0=vr0=vr0dc8meaabaqaciaacaGaaeqabaqabeGadaaakeaaiiGacqWFjpWDdaWgaaWcbaGaemitaWeabeaakiabcIcaOiabdsha0jabcMcaPiabg2da9iGbcwgaLjabcIha4jabcchaWnaabmaabaGaeyOeI0Yaa8qmaeaacqWGNbWzcqGGOaakcqWGmbatcqGGOaakcqWFepaDcqGGPaqkcqGGPaqkaSqaaiabdsha0jabgkHiTiabdsfaunaaBaaameaacqWGmbataeqaaaWcbaGaemiDaqhaniabgUIiYdGccqqGGaaicqWGKbazcqWFepaDaiaawIcacaGLPaaacqGGUaGlaaa@4F8E@

This expression sums the risk of surviving a mortality hazard that may change over the duration of the larval stage as the numbers of larvae vary (*τ *is a dummy integration variable). Differentiating this expression a further DDE is obtained,

dωL(t)dt=ωL(t)[g(L(t−TL))−g(L(t))],
 MathType@MTEF@5@5@+=feaafiart1ev1aaatCvAUfKttLearuWrP9MDH5MBPbIqV92AaeXatLxBI9gBaebbnrfifHhDYfgasaacH8akY=wiFfYdH8Gipec8Eeeu0xXdbba9frFj0=OqFfea0dXdd9vqai=hGuQ8kuc9pgc9s8qqaq=dirpe0xb9q8qiLsFr0=vr0=vr0dc8meaabaqaciaacaGaaeqabaqabeGadaaakeaadaWcaaqaaiabdsgaKHGaciab=L8a3naaBaaaleaacqWGmbataeqaaOGaeiikaGIaemiDaqNaeiykaKcabaGaemizaqMaemiDaqhaaiabg2da9iab=L8a3naaBaaaleaacqWGmbataeqaaOGaeiikaGIaemiDaqNaeiykaKYaamWaaeaacqWGNbWzcqGGOaakcqWGmbatcqGGOaakcqWG0baDcqGHsislcqWGubavdaWgaaWcbaGaemitaWeabeaakiabcMcaPiabcMcaPiabgkHiTiabdEgaNjabcIcaOiabdYeamjabcIcaOiabdsha0jabcMcaPiabcMcaPaGaay5waiaaw2faaiabcYcaSaaa@5433@

completing the set of three equations needed to specify Model 1, the system in the absence of *Plasmodium*.

### Model 2, Mosquito with Plasmodium

Now introduce the *Plasmodium *in the way sketched in Figure 1. Exposed and infected mosquitoes are allowed to reproduce, though not necessarily at the same rate as susceptible insects, so terms in Model 1 involving *λ*_*S *_*S*(.) are replaced by *λ*_*S *_*S*(.) + *λ*_*E *_*E*(.) + *λ*_*I *_*S*(.). Into the susceptible adult balance equation a term – *a c x*(*t*)*S*(*t*) is introduced which represents the mosquitoes that feed on infected humans and pick up the malaria parasite. In this model it is assumed that that the dynamics of malaria in its human host can be ignored and the fraction of infected people remains constant at *x*.

This same term of course represents input into the exposed stage, from which there are two losses, density-independent mortality at rate *μ*_*E *_and maturation into the infectious stage. This latter quantity is simply the recruitment to the exposed stage *T*_*E *_days ago multiplied by the probability of living through the stage. The transfer out of the exposed stage is the recruitment to the infectious stage from which there is a single loss term representing density-independent mortality at rate *μ*_*I*_.

Putting this all together a system of five DDEs is obtained,

dL(t)dt=[λSS(t−TO)+λEE(t−TO)+λII(t−TO)]θO−[λSS(t−TO−TL)+λEE(t−TO−TL)+λII(t−TO−TL)]θOθLωL(t)−μLL(t)−g(L(t))L(t),
 MathType@MTEF@5@5@+=feaafiart1ev1aaatCvAUfKttLearuWrP9MDH5MBPbIqV92AaeXatLxBI9gBaebbnrfifHhDYfgasaacH8akY=wiFfYdH8Gipec8Eeeu0xXdbba9frFj0=OqFfea0dXdd9vqai=hGuQ8kuc9pgc9s8qqaq=dirpe0xb9q8qiLsFr0=vr0=vr0dc8meaabaqaciaacaGaaeqabaqabeGadaaakeaafaqaaeGabaaabaWaaSaaaeaacqWGKbazcqWGmbatcqGGOaakcqWG0baDcqGGPaqkaeaacqWGKbazcqWG0baDaaGaeyypa0ZaamWaaeaaiiGacqWF7oaBdaWgaaWcbaGaem4uamfabeaakiabdofatjabcIcaOiabdsha0jabgkHiTiabdsfaunaaBaaaleaacqWGpbWtaeqaaOGaeiykaKIaey4kaSIae83UdW2aaSbaaSqaaiabdweafbqabaGccqWGfbqrcqGGOaakcqWG0baDcqGHsislcqWGubavdaWgaaWcbaGaem4ta8eabeaakiabcMcaPiabgUcaRiab=T7aSnaaBaaaleaacqWGjbqsaeqaaOGaemysaKKaeiikaGIaemiDaqNaeyOeI0Iaemivaq1aaSbaaSqaaiabd+eapbqabaGccqGGPaqkaiaawUfacaGLDbaacqWF4oqCdaWgaaWcbaGaem4ta8eabeaaaOqaauaabiqaceaaaeaacaWLjaGaeyOeI0YaamWaaeaacqWF7oaBdaWgaaWcbaGaem4uamfabeaakiabdofatjabcIcaOiabdsha0jabgkHiTiabdsfaunaaBaaaleaacqWGpbWtaeqaaOGaeyOeI0Iaemivaq1aaSbaaSqaaiabdYeambqabaGccqGGPaqkcqGHRaWkcqWF7oaBdaWgaaWcbaGaemyraueabeaakiabdweafjabcIcaOiabdsha0jabgkHiTiabdsfaunaaBaaaleaacqWGpbWtaeqaaOGaeyOeI0Iaemivaq1aaSbaaSqaaiabdYeambqabaGccqGGPaqkcqGHRaWkcqWF7oaBdaWgaaWcbaGaemysaKeabeaakiabdMeajjabcIcaOiabdsha0jabgkHiTiabdsfaunaaBaaaleaacqWGpbWtaeqaaOGaeyOeI0Iaemivaq1aaSbaaSqaaiabdYeambqabaGccqGGPaqkaiaawUfacaGLDbaacqWF4oqCdaWgaaWcbaGaem4ta8eabeaakiab=H7aXnaaBaaaleaacqWGmbataeqaaOGae8xYdC3aaSbaaSqaaiabdYeambqabaGccqGGOaakcqWG0baDcqGGPaqkaeaacqGHsislcqWF8oqBdaWgaaWcbaGaemitaWeabeaakiabdYeamjabcIcaOiabdsha0jabcMcaPiabgkHiTiabdEgaNjabcIcaOiabdYeamjabcIcaOiabdsha0jabcMcaPiabcMcaPiabdYeamjabcIcaOiabdsha0jabcMcaPaaacqGGSaalaaaaaa@AF1C@

dS(t)dt=[λSS(t−TO−TL−TP)+λEE(t−TO−TL−TP)+λII(t−TO−TL−TP)]×θOθLωL(t−TP)θP−μSS(t)−acxS(t),
 MathType@MTEF@5@5@+=feaafiart1ev1aaatCvAUfKttLearuWrP9MDH5MBPbIqV92AaeXatLxBI9gBaebbnrfifHhDYfgasaacH8akY=wiFfYdH8Gipec8Eeeu0xXdbba9frFj0=OqFfea0dXdd9vqai=hGuQ8kuc9pgc9s8qqaq=dirpe0xb9q8qiLsFr0=vr0=vr0dc8meaabaqaciaacaGaaeqabaqabeGadaaakeaafaqabeGabaaabaWaaSaaaeaacqWGKbazcqWGtbWucqGGOaakcqWG0baDcqGGPaqkaeaacqWGKbazcqWG0baDaaGaeyypa0ZaamWaaeaaiiGacqWF7oaBdaWgaaWcbaGaem4uamfabeaakiabdofatjabcIcaOiabdsha0jabgkHiTiabdsfaunaaBaaaleaacqWGpbWtaeqaaOGaeyOeI0Iaemivaq1aaSbaaSqaaiabdYeambqabaGccqGHsislcqWGubavdaWgaaWcbaGaemiuaafabeaakiabcMcaPiabgUcaRiab=T7aSnaaBaaaleaacqWGfbqraeqaaOGaemyrauKaeiikaGIaemiDaqNaeyOeI0Iaemivaq1aaSbaaSqaaiabd+eapbqabaGccqGHsislcqWGubavdaWgaaWcbaGaemitaWeabeaakiabgkHiTiabdsfaunaaBaaaleaacqWGqbauaeqaaOGaeiykaKIaey4kaSIae83UdW2aaSbaaSqaaiabdMeajbqabaGccqWGjbqscqGGOaakcqWG0baDcqGHsislcqWGubavdaWgaaWcbaGaem4ta8eabeaakiabgkHiTiabdsfaunaaBaaaleaacqWGmbataeqaaOGaeyOeI0Iaemivaq1aaSbaaSqaaiabdcfaqbqabaGccqGGPaqkaiaawUfacaGLDbaacqGHxdaTaeaacqWF4oqCdaWgaaWcbaGaem4ta8eabeaakiab=H7aXnaaBaaaleaacqWGmbataeqaaOGae8xYdC3aaSbaaSqaaiabdYeambqabaGccqGGOaakcqWG0baDcqGHsislcqWGubavdaWgaaWcbaGaemiuaafabeaakiabcMcaPiab=H7aXnaaBaaaleaacqWGqbauaeqaaOGaeyOeI0Iae8hVd02aaSbaaSqaaiabdofatbqabaGccqWGtbWucqGGOaakcqWG0baDcqGGPaqkcqGHsislcqWGHbqycqWGJbWycqWG4baEcqWGtbWucqGGOaakcqWG0baDcqGGPaqkaaGaeiilaWcaaa@9692@

dωL(t)dt=ωL(t)[g(L(t−TL))−g(L(t))],
 MathType@MTEF@5@5@+=feaafiart1ev1aaatCvAUfKttLearuWrP9MDH5MBPbIqV92AaeXatLxBI9gBaebbnrfifHhDYfgasaacH8akY=wiFfYdH8Gipec8Eeeu0xXdbba9frFj0=OqFfea0dXdd9vqai=hGuQ8kuc9pgc9s8qqaq=dirpe0xb9q8qiLsFr0=vr0=vr0dc8meaabaqaciaacaGaaeqabaqabeGadaaakeaadaWcaaqaaiabdsgaKHGaciab=L8a3naaBaaaleaacqWGmbataeqaaOGaeiikaGIaemiDaqNaeiykaKcabaGaemizaqMaemiDaqhaaiabg2da9iab=L8a3naaBaaaleaacqWGmbataeqaaOGaeiikaGIaemiDaqNaeiykaKYaamWaaeaacqWGNbWzcqGGOaakcqWGmbatcqGGOaakcqWG0baDcqGHsislcqWGubavdaWgaaWcbaGaemitaWeabeaakiabcMcaPiabcMcaPiabgkHiTiabdEgaNjabcIcaOiabdYeamjabcIcaOiabdsha0jabcMcaPiabcMcaPaGaay5waiaaw2faaiabcYcaSaaa@5433@

dE(t)dt=acxS(t)−acxS(t−TE)θE−μEE(t),
 MathType@MTEF@5@5@+=feaafiart1ev1aaatCvAUfKttLearuWrP9MDH5MBPbIqV92AaeXatLxBI9gBaebbnrfifHhDYfgasaacH8akY=wiFfYdH8Gipec8Eeeu0xXdbba9frFj0=OqFfea0dXdd9vqai=hGuQ8kuc9pgc9s8qqaq=dirpe0xb9q8qiLsFr0=vr0=vr0dc8meaabaqaciaacaGaaeqabaqabeGadaaakeaadaWcaaqaaiabdsgaKjabdweafjabcIcaOiabdsha0jabcMcaPaqaaiabdsgaKjabdsha0baacqGH9aqpcqWGHbqycqWGJbWycqWG4baEcqWGtbWucqGGOaakcqWG0baDcqGGPaqkcqGHsislcqWGHbqycqWGJbWycqWG4baEcqWGtbWucqGGOaakcqWG0baDcqGHsislcqWGubavdaWgaaWcbaGaemyraueabeaakiabcMcaPGGaciab=H7aXnaaBaaaleaacqWGfbqraeqaaOGaeyOeI0Iae8hVd02aaSbaaSqaaiabdweafbqabaGccqWGfbqrcqGGOaakcqWG0baDcqGGPaqkcqGGSaalaaa@572C@

dI(t)dt=acxS(t−TE)θE−μII(t).
 MathType@MTEF@5@5@+=feaafiart1ev1aaatCvAUfKttLearuWrP9MDH5MBPbIqV92AaeXatLxBI9gBaebbnrfifHhDYfgasaacH8akY=wiFfYdH8Gipec8Eeeu0xXdbba9frFj0=OqFfea0dXdd9vqai=hGuQ8kuc9pgc9s8qqaq=dirpe0xb9q8qiLsFr0=vr0=vr0dc8meaabaqaciaacaGaaeqabaqabeGadaaakeaadaWcaaqaaiabdsgaKjabdMeajjabcIcaOiabdsha0jabcMcaPaqaaiabdsgaKjabdsha0baacqGH9aqpcqWGHbqycqWGJbWycqWG4baEcqWGtbWucqGGOaakcqWG0baDcqGHsislcqWGubavdaWgaaWcbaGaemyraueabeaakiabcMcaPGGaciab=H7aXnaaBaaaleaacqWGfbqraeqaaOGaeyOeI0Iae8hVd02aaSbaaSqaaiabdMeajbqabaGccqWGjbqscqGGOaakcqWG0baDcqGGPaqkcqGGUaGlaaa@4DF6@

It will sometimes be useful to study the dynamics of the total number of adult mosquitoes: *N*(*t*) = *S*(*t*) + *E*(*t*) + *I*(*t*); summing eqns 2b, 2d & 2e,

dN(t)dt=[λSS(t−TO−TL−TP)+λEE(t−TO−TL−TP)+λII(t−TO−TL−TP)]×θOθLωL(t−TP)θP−[μSS(t)+μEE(t)+μII(t)]
 MathType@MTEF@5@5@+=feaafiart1ev1aaatCvAUfKttLearuWrP9MDH5MBPbIqV92AaeXatLxBI9gBaebbnrfifHhDYfgasaacH8akY=wiFfYdH8Gipec8Eeeu0xXdbba9frFj0=OqFfea0dXdd9vqai=hGuQ8kuc9pgc9s8qqaq=dirpe0xb9q8qiLsFr0=vr0=vr0dc8meaabaqaciaacaGaaeqabaqabeGadaaakeaafaqabeGabaaabaWaaSaaaeaacqWGKbazcqWGobGtcqGGOaakcqWG0baDcqGGPaqkaeaacqWGKbazcqWG0baDaaGaeyypa0ZaamWaaeaaiiGacqWF7oaBdaWgaaWcbaGaem4uamfabeaakiabdofatjabcIcaOiabdsha0jabgkHiTiabdsfaunaaBaaaleaacqWGpbWtaeqaaOGaeyOeI0Iaemivaq1aaSbaaSqaaiabdYeambqabaGccqGHsislcqWGubavdaWgaaWcbaGaemiuaafabeaakiabcMcaPiabgUcaRiab=T7aSnaaBaaaleaacqWGfbqraeqaaOGaemyrauKaeiikaGIaemiDaqNaeyOeI0Iaemivaq1aaSbaaSqaaiabd+eapbqabaGccqGHsislcqWGubavdaWgaaWcbaGaemitaWeabeaakiabgkHiTiabdsfaunaaBaaaleaacqWGqbauaeqaaOGaeiykaKIaey4kaSIae83UdW2aaSbaaSqaaiabdMeajbqabaGccqWGjbqscqGGOaakcqWG0baDcqGHsislcqWGubavdaWgaaWcbaGaem4ta8eabeaakiabgkHiTiabdsfaunaaBaaaleaacqWGmbataeqaaOGaeyOeI0Iaemivaq1aaSbaaSqaaiabdcfaqbqabaGccqGGPaqkaiaawUfacaGLDbaacqGHxdaTaeaacqWF4oqCdaWgaaWcbaGaem4ta8eabeaakiab=H7aXnaaBaaaleaacqWGmbataeqaaOGae8xYdC3aaSbaaSqaaiabdYeambqabaGccqGGOaakcqWG0baDcqGHsislcqWGubavdaWgaaWcbaGaemiuaafabeaakiabcMcaPiab=H7aXnaaBaaaleaacqWGqbauaeqaaOGaeyOeI0YaamWaaeaacqWF8oqBdaWgaaWcbaGaem4uamfabeaakiabdofatjabcIcaOiabdsha0jabcMcaPiabgUcaRiab=X7aTnaaBaaaleaacqWGfbqraeqaaOGaemyrauKaeiikaGIaemiDaqNaeiykaKIaey4kaSIae8hVd02aaSbaaSqaaiabdMeajbqabaGccqWGjbqscqGGOaakcqWG0baDcqGGPaqkaiaawUfacaGLDbaaaaaaaa@9E7C@

### Model 3, Mosquito with Plasmodium and a simplified human stage

As discussed above, no pretence is made here to model with any great realism the dynamics of the pathogen in its human stage. However, it is shown how in principle the dynamics of the vector and definitive host can be coupled.

The representation of the mosquito dynamics is identical to that in model 2 except that *x *is replaced by *x*(*t*) in eqns. 2c-f because the fraction of humans infected now changes over time (these equations will not be written down again but referred to in the context of this model as eqns. 3a-f). It is assumed that the fraction of people infected increases as mosquitoes attack humans at a rate *a *and successfully infect them with probability *b*. The simplest assumption is that humans immediately become capable of transmitting malaria and recover at a constant rate *r*. This leads to the equation

dx(t)dt=abI(t)H(1−x(t))−rx(t),
 MathType@MTEF@5@5@+=feaafiart1ev1aaatCvAUfKttLearuWrP9MDH5MBPbIqV92AaeXatLxBI9gBaebbnrfifHhDYfgasaacH8akY=wiFfYdH8Gipec8Eeeu0xXdbba9frFj0=OqFfea0dXdd9vqai=hGuQ8kuc9pgc9s8qqaq=dirpe0xb9q8qiLsFr0=vr0=vr0dc8meaabaqaciaacaGaaeqabaqabeGadaaakeaadaWcaaqaaiabdsgaKjabdIha4jabcIcaOiabdsha0jabcMcaPaqaaiabdsgaKjabdsha0baacqGH9aqpcqWGHbqycqWGIbGydaWcaaqaaiabdMeajjabcIcaOiabdsha0jabcMcaPaqaaiabdIeaibaacqGGOaakcqaIXaqmcqGHsislcqWG4baEcqGGOaakcqWG0baDcqGGPaqkcqGGPaqkcqGHsislcqWGYbGCcqWG4baEcqGGOaakcqWG0baDcqGGPaqkcqGGSaalaaa@4E71@

where *H *is the total number or density of humans, assumed to be a constant. But time delays associated with the gap between infection and harbouring transmissible gametocytes (suppose this last *T*_*α *_days), and between the onset of infectiousness and clearance of the disease by the immune system (define this as lasting *T*_*β *_days), can also be included. If no human mortality is assumed then

dx(t)dt=abI(t−Tα)H(1−x(t−Tα))−abI(t−Tα−Tβ)H(1−x(t−Tα−Tβ)).
 MathType@MTEF@5@5@+=feaafiart1ev1aaatCvAUfKttLearuWrP9MDH5MBPbIqV92AaeXatLxBI9gBaebbnrfifHhDYfgasaacH8akY=wiFfYdH8Gipec8Eeeu0xXdbba9frFj0=OqFfea0dXdd9vqai=hGuQ8kuc9pgc9s8qqaq=dirpe0xb9q8qiLsFr0=vr0=vr0dc8meaabaqaciaacaGaaeqabaqabeGadaaakeaadaWcaaqaaiabdsgaKjabdIha4jabcIcaOiabdsha0jabcMcaPaqaaiabdsgaKjabdsha0baacqGH9aqpcqWGHbqycqWGIbGydaWcaaqaaiabdMeajjabcIcaOiabdsha0jabgkHiTiabdsfaunaaBaaaleaaiiGacqWFXoqyaeqaaOGaeiykaKcabaGaemisaGeaaiabcIcaOiabigdaXiabgkHiTiabdIha4jabcIcaOiabdsha0jabgkHiTiabdsfaunaaBaaaleaacqWFXoqyaeqaaOGaeiykaKIaeiykaKIaeyOeI0IaemyyaeMaemOyai2aaSaaaeaacqWGjbqscqGGOaakcqWG0baDcqGHsislcqWGubavdaWgaaWcbaGae8xSdegabeaakiabgkHiTiabdsfaunaaBaaaleaacqWFYoGyaeqaaOGaeiykaKcabaGaemisaGeaaiabcIcaOiabigdaXiabgkHiTiabdIha4jabcIcaOiabdsha0jabgkHiTiabdsfaunaaBaaaleaacqWFXoqyaeqaaOGaeyOeI0Iaemivaq1aaSbaaSqaaiab=j7aIbqabaGccqGGPaqkcqGGPaqkcqGGUaGlaaa@703A@

### Mosquito density dependence

Models 1 – 3 are now fully specified with the exception of the function describing mosquito density dependence. Here the very simplest assumption, linear competition, is assumed

*g*(*L*(*t*)) = *γL*(*t*).

This is equivalent to assuming Lotka-Volterra competition in classical ecological theory, though the time lags in the system make the behaviour of the model more akin to its discrete-time equivalent, the Ricker process [27].

Note that *L*(*t*) is the density of female mosquitoes whilst all reasonable models of larval competition would assume males and females have the same or at least a similar effect. To keep things simple an equal sex ratio is assumed, as well as equal male and female contributions to competition (so that the number of mosquitoes influencing mortality through competition is 2 *L*(*t*)), and the multiplier 2 is subsumed within the parameter *γ*.

## Analysis: statics

It is argued that the main function of the models developed here is to study the dynamics of mosquito populations, and their response to potential perturbations. Nevertheless it is important to demonstrate the link with some of the classical static quantities that have been used by vector entomologists which is what is done in this section.

### Model 1, Mosquito with no Plasmodium

First ask whether the mosquito population can invade a habitat (or equivalently what mortality needs to be imposed on the vector before it is driven to extinction). At these threshold population levels negligible density-dependent mortality (*ω*_*L *_→ 1) can be assumed and hence fecundity must be sufficiently high to offset the different density-independent mortality factors. From eqn. 1b, population growth rates are only positive when

Λ=λSθoθLθPμS>1
 MathType@MTEF@5@5@+=feaafiart1ev1aaatCvAUfKttLearuWrP9MDH5MBPbIqV92AaeXatLxBI9gBaebbnrfifHhDYfgasaacH8akY=wiFfYdH8Gipec8Eeeu0xXdbba9frFj0=OqFfea0dXdd9vqai=hGuQ8kuc9pgc9s8qqaq=dirpe0xb9q8qiLsFr0=vr0=vr0dc8meaabaqaciaacaGaaeqabaqabeGadaaakeaacqqHBoatcqGH9aqpdaWcaaqaaGGaciab=T7aSnaaBaaaleaacqWGtbWuaeqaaOGae8hUde3aaSbaaSqaaiabd+gaVbqabaGccqWF4oqCdaWgaaWcbaGaemitaWeabeaakiab=H7aXnaaBaaaleaacqWGqbauaeqaaaGcbaGae8hVd02aaSbaaSqaaiabdofatbqabaaaaOGaeyOpa4JaeGymaedaaa@40CB@

which has a very simple interpretation. The numerator in eqn. 5 is the rate of production of *adult *mosquitoes – fecundity multiplied by the different probabilities of surviving the three juvenile stages – while 1/*μ*_*S *_is expected adult lifespan. The expression thus states the obvious fact that for a population to persist each female mosquito must at least replace itself, or that the number of adult female offspring (*Λ*) produced per female must be great than one. Substituting *θ*_*i *_= Exp[-*T*_*i*_*μ*_*i*_] for the different juvenile stages in eqn. 5 shows how the different stage durations and mortality components combine to determine population persistence. It can also be used to explore the effects of artificially increasing different mortality factors as part of a control programme.

At equilibrium the density dependent mortality must be sufficient to reduce the effective adult female offspring production to one. Thus it must impose a mortality such that the probability of survival is 1/*Λ *which allows us to calculate the equilibrium larval density (L*)

ωL=1Λ⇒exp⁡[−TLg(L∗)]=1Λ⇒g(L∗)=1TLln⁡Λ.
 MathType@MTEF@5@5@+=feaafiart1ev1aaatCvAUfKttLearuWrP9MDH5MBPbIqV92AaeXatLxBI9gBaebbnrfifHhDYfgasaacH8akY=wiFfYdH8Gipec8Eeeu0xXdbba9frFj0=OqFfea0dXdd9vqai=hGuQ8kuc9pgc9s8qqaq=dirpe0xb9q8qiLsFr0=vr0=vr0dc8meaabaqaciaacaGaaeqabaqabeGadaaakeaaiiGacqWFjpWDdaWgaaWcbaGaemitaWeabeaakiabg2da9maalaaabaGaeGymaedabaGaeu4MdWeaaiabgkDiElGbcwgaLjabcIha4jabcchaWjabcUfaBjabgkHiTiabdsfaunaaBaaaleaacqWGmbataeqaaOGaem4zaCMaeiikaGIaemitaW0aaWbaaSqabeaacqGHxiIkaaGccqGGPaqkcqGGDbqxcqGH9aqpdaWcaaqaaiabigdaXaqaaiabfU5ambaacqGHshI3cqWGNbWzcqGGOaakcqWGmbatdaahaaWcbeqaaiabgEHiQaaakiabcMcaPiabg2da9maalaaabaGaeGymaedabaGaemivaq1aaSbaaSqaaiabdYeambqabaaaaOGagiiBaWMaeiOBa4Maeu4MdWKaeiOla4caaa@59FD@

With the linear competition assumption L* = ln *Λ*/*γ T*_*L*_.

Of course, it is the density of adult mosquitoes that more concerns vector biologists, and this is simply calculated from eqn. 1a

S∗=L∗(μL+g(L∗))λSθO−μSθP
 MathType@MTEF@5@5@+=feaafiart1ev1aaatCvAUfKttLearuWrP9MDH5MBPbIqV92AaeXatLxBI9gBaebbnrfifHhDYfgasaacH8akY=wiFfYdH8Gipec8Eeeu0xXdbba9frFj0=OqFfea0dXdd9vqai=hGuQ8kuc9pgc9s8qqaq=dirpe0xb9q8qiLsFr0=vr0=vr0dc8meaabaqaciaacaGaaeqabaqabeGadaaakeaacqWGtbWudaahaaWcbeqaaiabgEHiQaaakiabg2da9maalaaabaGaemitaW0aaWbaaSqabeaacqGHxiIkaaGccqGGOaakiiGacqWF8oqBdaWgaaWcbaGaemitaWeabeaakiabgUcaRiabdEgaNjabcIcaOiabdYeamnaaCaaaleqabaGaey4fIOcaaOGaeiykaKIaeiykaKcabaGae83UdW2aaSbaaSqaaiabdofatbqabaGccqWF4oqCdaWgaaWcbaGaem4ta8eabeaakiabgkHiTmaalmaaleaacqWF8oqBdaWgaaadbaGaem4uamfabeaaaSqaaiab=H7aXnaaBaaameaacqWGqbauaeqaaaaaaaaaaa@4AA2@

though this is less amenable to a simple interpretation.

### Model 2, Mosquito with Plasmodium

#### Equilibria

Consider first how the inclusion of three different classes of adult mosquitoes affects the persistence conditions and equilibria derived in the preceding section. From eqn.2f it is straightforward to show that persistence requires

Λ¯=λ¯θoθLθPμ¯>1,
 MathType@MTEF@5@5@+=feaafiart1ev1aaatCvAUfKttLearuWrP9MDH5MBPbIqV92AaeXatLxBI9gBaebbnrfifHhDYfgasaacH8akY=wiFfYdH8Gipec8Eeeu0xXdbba9frFj0=OqFfea0dXdd9vqai=hGuQ8kuc9pgc9s8qqaq=dirpe0xb9q8qiLsFr0=vr0=vr0dc8meaabaqaciaacaGaaeqabaqabeGadaaakeGabaGjpiqbfU5amzaaraGaeyypa0ZaaSaaaeaaiiGacuWF7oaBgaqeaiab=H7aXnaaBaaaleaacqWGVbWBaeqaaOGae8hUde3aaSbaaSqaaiabdYeambqabaGccqWF4oqCdaWgaaWcbaGaemiuaafabeaaaOqaaiqb=X7aTzaaraaaaiabg6da+iabigdaXiabcYcaSaaa@3F61@

where λ¯
 MathType@MTEF@5@5@+=feaafiart1ev1aaatCvAUfKttLearuWrP9MDH5MBPbIqV92AaeXatLxBI9gBaebbnrfifHhDYfgasaacH8akY=wiFfYdH8Gipec8Eeeu0xXdbba9frFj0=OqFfea0dXdd9vqai=hGuQ8kuc9pgc9s8qqaq=dirpe0xb9q8qiLsFr0=vr0=vr0dc8meaabaqaciaacaGaaeqabaqabeGadaaakeaaiiGacuWF7oaBgaqeaaaa@2E7F@ is the average fecundity of an adult mosquito,

λ¯
 MathType@MTEF@5@5@+=feaafiart1ev1aaatCvAUfKttLearuWrP9MDH5MBPbIqV92AaeXatLxBI9gBaebbnrfifHhDYfgasaacH8akY=wiFfYdH8Gipec8Eeeu0xXdbba9frFj0=OqFfea0dXdd9vqai=hGuQ8kuc9pgc9s8qqaq=dirpe0xb9q8qiLsFr0=vr0=vr0dc8meaabaqaciaacaGaaeqabaqabeGadaaakeaaiiGacuWF7oaBgaqeaaaa@2E7F@ = *λ*_*S *_Pr(*S*) + *λ*_*I *_Pr(*I*) + *λ*_*E *_Pr(*E*),

and Pr(*i*) denotes the proportion of adults in stage *i*. The average adult mortality, μ¯
 MathType@MTEF@5@5@+=feaafiart1ev1aaatCvAUfKttLearuWrP9MDH5MBPbIqV92AaeXatLxBI9gBaebbnrfifHhDYfgasaacH8akY=wiFfYdH8Gipec8Eeeu0xXdbba9frFj0=OqFfea0dXdd9vqai=hGuQ8kuc9pgc9s8qqaq=dirpe0xb9q8qiLsFr0=vr0=vr0dc8meaabaqaciaacaGaaeqabaqabeGadaaakeaaiiGacuWF8oqBgaqeaaaa@2E81@, is calculated in the same way. From eqn. 2d and 2e the ratio of adults in the three stages is

S:E:I=1:acx(1−θE)μE:acxθEμI,
 MathType@MTEF@5@5@+=feaafiart1ev1aaatCvAUfKttLearuWrP9MDH5MBPbIqV92AaeXatLxBI9gBaebbnrfifHhDYfgasaacH8akY=wiFfYdH8Gipec8Eeeu0xXdbba9frFj0=OqFfea0dXdd9vqai=hGuQ8kuc9pgc9s8qqaq=dirpe0xb9q8qiLsFr0=vr0=vr0dc8meaabaqaciaacaGaaeqabaqabeGadaaakeaacqWGtbWucqGG6aGocqWGfbqrcqGG6aGocqWGjbqscqGH9aqpcqaIXaqmcqGG6aGodaWcaaqaaiabdggaHjabdogaJjabdIha4jabcIcaOiabigdaXiabgkHiTGGaciab=H7aXnaaBaaaleaacqWGfbqraeqaaOGaeiykaKcabaGae8hVd02aaSbaaSqaaiabdweafbqabaaaaOGaeiOoaOZaaSaaaeaacqWGHbqycqWGJbWycqWG4baEcqWF4oqCdaWgaaWcbaGaemyraueabeaaaOqaaiab=X7aTnaaBaaaleaacqWGjbqsaeqaaaaakiabcYcaSaaa@4EA0@

from which the three fractions can be calculated. The equilibrium density of larvae remain the same but using the new definition of Λ¯
 MathType@MTEF@5@5@+=feaafiart1ev1aaatCvAUfKttLearuWrP9MDH5MBPbIqV92AaeXatLxBI9gBaebbnrfifHhDYfgasaacH8akY=wiFfYdH8Gipec8Eeeu0xXdbba9frFj0=OqFfea0dXdd9vqai=hGuQ8kuc9pgc9s8qqaq=dirpe0xb9q8qiLsFr0=vr0=vr0dc8meaabaqaciaacaGaaeqabaqabeGadaaakeaacuqHBoatgaqeaaaa@2E39@, while the total density of adults is

N∗=L∗(μL+g(L∗))λ¯θO−μ¯θP.
 MathType@MTEF@5@5@+=feaafiart1ev1aaatCvAUfKttLearuWrP9MDH5MBPbIqV92AaeXatLxBI9gBaebbnrfifHhDYfgasaacH8akY=wiFfYdH8Gipec8Eeeu0xXdbba9frFj0=OqFfea0dXdd9vqai=hGuQ8kuc9pgc9s8qqaq=dirpe0xb9q8qiLsFr0=vr0=vr0dc8meaabaqaciaacaGaaeqabaqabeGadaaakeaacqWGobGtdaahaaWcbeqaaiabgEHiQaaakiabg2da9maalaaabaGaemitaW0aaWbaaSqabeaacqGHxiIkaaGccqGGOaakiiGacqWF8oqBdaWgaaWcbaGaemitaWeabeaakiabgUcaRiabdEgaNjabcIcaOiabdYeamnaaCaaaleqabaGaey4fIOcaaOGaeiykaKIaeiykaKcabaGaf83UdWMbaebacqWF4oqCdaWgaaWcbaGaem4ta8eabeaakiabgkHiTmaalmaaleaacuWF8oqBgaqeaaqaaiab=H7aXnaaBaaameaacqWGqbauaeqaaaaaaaGccqGGUaGlaaa@48EA@

Again, the numbers in each adult class can be calculated using eqn. 8.

It is often useful to write down a term for the numbers of adult mosquitoes emerging per unit time, *ε*(*t*); from eqns. 2b & 9,

*ε*(*t*) = μ¯
 MathType@MTEF@5@5@+=feaafiart1ev1aaatCvAUfKttLearuWrP9MDH5MBPbIqV92AaeXatLxBI9gBaebbnrfifHhDYfgasaacH8akY=wiFfYdH8Gipec8Eeeu0xXdbba9frFj0=OqFfea0dXdd9vqai=hGuQ8kuc9pgc9s8qqaq=dirpe0xb9q8qiLsFr0=vr0=vr0dc8meaabaqaciaacaGaaeqabaqabeGadaaakeaaiiGacuWF8oqBgaqeaaaa@2E81@Λ¯
 MathType@MTEF@5@5@+=feaafiart1ev1aaatCvAUfKttLearuWrP9MDH5MBPbIqV92AaeXatLxBI9gBaebbnrfifHhDYfgasaacH8akY=wiFfYdH8Gipec8Eeeu0xXdbba9frFj0=OqFfea0dXdd9vqai=hGuQ8kuc9pgc9s8qqaq=dirpe0xb9q8qiLsFr0=vr0=vr0dc8meaabaqaciaacaGaaeqabaqabeGadaaakeaacuqHBoatgaqeaaaa@2E39@*ω*_*L*_(*t *- *T*_*P*_) *N *(*t *- *T*_*O *_- *T*_*L *_- *T*_*P*_).

At equilibrium, ε* = μ¯
 MathType@MTEF@5@5@+=feaafiart1ev1aaatCvAUfKttLearuWrP9MDH5MBPbIqV92AaeXatLxBI9gBaebbnrfifHhDYfgasaacH8akY=wiFfYdH8Gipec8Eeeu0xXdbba9frFj0=OqFfea0dXdd9vqai=hGuQ8kuc9pgc9s8qqaq=dirpe0xb9q8qiLsFr0=vr0=vr0dc8meaabaqaciaacaGaaeqabaqabeGadaaakeaaiiGacuWF8oqBgaqeaaaa@2E81@N*, the numbers emerging equal the numbers dying.

#### Epidemiological statics

A series of quantities frequently used in the mosquito literature can now be derived and compared to the forms given by Smith & McKenzie (2004) in their review of mosquito epidemiological statics and dynamics (see in particular their Table 2; note that they include a parameter to describe the fraction of mosquitoes that feed on non-human hosts which here is subsumed in the feeding rate, *a*).

The sporozoite rate is the fraction of mosquitoes that are infectious to humans, and is easily derived from the ratios in eqn. 8,

Sporozoite Rate (SR)=acxθEμI+acx[θE+(1−θE)μIμE].
 MathType@MTEF@5@5@+=feaafiart1ev1aaatCvAUfKttLearuWrP9MDH5MBPbIqV92AaeXatLxBI9gBaebbnrfifHhDYfgasaacH8akY=wiFfYdH8Gipec8Eeeu0xXdbba9frFj0=OqFfea0dXdd9vqai=hGuQ8kuc9pgc9s8qqaq=dirpe0xb9q8qiLsFr0=vr0=vr0dc8meaabaqaciaacaGaaeqabaqabeGadaaakeaacqqGtbWucqqGWbaCcqqGVbWBcqqGYbGCcqqGVbWBcqqG6bGEcqqGVbWBcqqGPbqAcqqG0baDcqqGLbqzcqqGGaaicqqGsbGucqqGHbqycqqG0baDcqqGLbqzcqqGGaaicqqGOaakcqqGtbWucqqGsbGucqqGPaqkiiaacqWF9aqpdaWcaaqaaiabdggaHjabdogaJjabdIha4HGaciab+H7aXnaaBaaaleaacqWGfbqraeqaaaGcbaGae4hVd02aaSbaaSqaaiabdMeajbqabaGccqGHRaWkcqWGHbqycqWGJbWycqWG4baEdaWadaqaaiab+H7aXnaaBaaaleaacqWGfbqraeqaaOGaey4kaSIaeiikaGIaeGymaeJaeyOeI0Iae4hUde3aaSbaaSqaaiabdweafbqabaGccqGGPaqkdaWcdaWcbaGae4hVd02aaSbaaWqaaiabdMeajbqabaaaleaacqGF8oqBdaWgaaadbaGaemyraueabeaaaaaakiaawUfacaGLDbaaaaGaeiOla4caaa@68B3@

If constant adult mortality is assumed then the term in square brackets in the denominator disappears and the classical form is obtained (Smith & McKenzie 2004).

The Lifetime Transmission Potential is the number of cases of malaria a mosquito can be expected to transmit during its complete lifetime. It is the product of the probability of becoming infectious, the average number of times an infectious mosquito will feed, and the efficiency of transmission from mosquito to human. The probability that a newly emerged female becomes infected before it dies is *acx*/(*acx*+*μ*_*S*_) and the probability it goes on to survive the exposed period is *θ*_*E*_. Once infectious it will live for on average 1/*μ*_*I *_days during which time it will feed at rate *a *transmitting the infection with probability *b*. Putting this together

Lifetime Transmission Potential (LTP)=a2bcxθEμI(μS+acx),
 MathType@MTEF@5@5@+=feaafiart1ev1aaatCvAUfKttLearuWrP9MDH5MBPbIqV92AaeXatLxBI9gBaebbnrfifHhDYfgasaacH8akY=wiFfYdH8Gipec8Eeeu0xXdbba9frFj0=OqFfea0dXdd9vqai=hGuQ8kuc9pgc9s8qqaq=dirpe0xb9q8qiLsFr0=vr0=vr0dc8meaabaqaciaacaGaaeqabaqabeGadaaakeaacqqGmbatcqqGPbqAcqqGMbGzcqqGLbqzcqqG0baDcqqGPbqAcqqGTbqBcqqGLbqzcqqGGaaicqqGubavcqqGYbGCcqqGHbqycqqGUbGBcqqGZbWCcqqGTbqBcqqGPbqAcqqGZbWCcqqGZbWCcqqGPbqAcqqGVbWBcqqGUbGBcqqGGaaicqqGqbaucqqGVbWBcqqG0baDcqqGLbqzcqqGUbGBcqqG0baDcqqGPbqAcqqGHbqycqqGSbaBcqqGGaaicqqGOaakcqqGmbatcqqGubavcqqGqbaucqqGPaqkiiaacqWF9aqpdaWcaaqaaiabdggaHnaaCaaaleqabaGaeGOmaidaaOGaemOyaiMaem4yamMaemiEaGhcciGae4hUde3aaSbaaSqaaiabdweafbqabaaakeaacqGF8oqBdaWgaaWcbaGaemysaKeabeaakmaabmaabaGae4hVd02aaSbaaSqaaiabdofatbqabaGccqGHRaWkcqWGHbqycqWGJbWycqWG4baEaiaawIcacaGLPaaaaaGaeiilaWcaaa@735E@

which reduces to the form in Smith & Mckenzie (2004) when mortality is constant throughout the adult stage.

The Entomological Inoculation Rate is the number of potentially infectious bites received per human per day. It is simply the total rate of feeding by infectious mosquitoes, *aI*(*t*), divided by the number of humans, *H*, which is assumed to be constant. At equilibrium the number of infectious mosquitoes is the total number of adult mosquitoes (N*) multiplied by the sporozoite rate. Thus

Entomological Inoculation Rate (EIR) =aN∗HSR=N∗Ha2cxθEμI+acx[θE+(1−θE)μIμE]
 MathType@MTEF@5@5@+=feaafiart1ev1aaatCvAUfKttLearuWrP9MDH5MBPbIqV92AaeXatLxBI9gBaebbnrfifHhDYfgasaacH8akY=wiFfYdH8Gipec8Eeeu0xXdbba9frFj0=OqFfea0dXdd9vqai=hGuQ8kuc9pgc9s8qqaq=dirpe0xb9q8qiLsFr0=vr0=vr0dc8meaabaqaciaacaGaaeqabaqabeGadaaakeaacqqGfbqrcqqGUbGBcqqG0baDcqqGVbWBcqqGTbqBcqqGVbWBcqqGSbaBcqqGVbWBcqqGNbWzcqqGPbqAcqqGJbWycqqGHbqycqqGSbaBcqqGGaaicqqGjbqscqqGUbGBcqqGVbWBcqqGJbWycqqG1bqDcqqGSbaBcqqGHbqycqqG0baDcqqGPbqAcqqGVbWBcqqGUbGBcqqGGaaicqqGsbGucqqGHbqycqqG0baDcqqGLbqzcqqGGaaicqqGOaakcqqGfbqrcqqGjbqscqqGsbGucqqGPaqkcqqGGaaicqGH9aqpcqWGHbqydaWcaaqaaiabd6eaonaaCaaaleqabaGaey4fIOcaaaGcbaGaemisaGeaaiabbofatjabbkfasjabg2da9maalaaabaGaemOta40aaWbaaSqabeaacqGHxiIkaaaakeaacqWGibasaaWaaSaaaeaacqWGHbqydaahaaWcbeqaaiabikdaYaaakiabdogaJjabdIha4HGaciab=H7aXnaaBaaaleaacqWGfbqraeqaaaGcbaGae8hVd02aaSbaaSqaaiabdMeajbqabaGccqGHRaWkcqWGHbqycqWGJbWycqWG4baEdaWadaqaaiab=H7aXnaaBaaaleaacqWGfbqraeqaaOGaey4kaSIaeiikaGIaeGymaeJaeyOeI0Iae8hUde3aaSbaaSqaaiabdweafbqabaGccqGGPaqkdaWcdaWcbaGae8hVd02aaSbaaWqaaiabdMeajbqabaaaleaacqWF8oqBdaWgaaadbaGaemyraueabeaaaaaakiaawUfacaGLDbaaaaaaaa@89A1@

which reduces to the form in Smith & McKenzie (2004) when mortality is constant throughout the adult stage. [Smith & McKenzie also derive the EIR as the product of the emergence rate of mosquitoes and the Lifetime Transmission Potential. This is equivalent when mortality is uniform throughout the adult stage (as they assumed) but not with variable mortality when the probability an individual becomes infectious is not the same as the fraction of infectious individuals.]

The Vectorial Capacity can be defined [28] as the total number of potentially infectious bites that arise when a single infected human is introduced for one day into a system where *Plasmodium *is currently absent. If it is assumed that mosquito densities are at equilibrium then the number of bites that that individual will suffer is aN*/*H *of which *c *will lead to successful infection. The probability that a successfully infected individual will survive the exposed period is *θ*_*E *_after which it will live for on average for 1/*μ*_*I *_days during which time it will feed at rate *a*. Thus

Vectorial Capacity (VC)=N∗Ha2cθEμI
 MathType@MTEF@5@5@+=feaafiart1ev1aaatCvAUfKttLearuWrP9MDH5MBPbIqV92AaeXatLxBI9gBaebbnrfifHhDYfgasaacH8akY=wiFfYdH8Gipec8Eeeu0xXdbba9frFj0=OqFfea0dXdd9vqai=hGuQ8kuc9pgc9s8qqaq=dirpe0xb9q8qiLsFr0=vr0=vr0dc8meaabaqaciaacaGaaeqabaqabeGadaaakeaacqqGwbGvcqqGLbqzcqqGJbWycqqG0baDcqqGVbWBcqqGYbGCcqqGPbqAcqqGHbqycqqGSbaBcqqGGaaicqqGdbWqcqqGHbqycqqGWbaCcqqGHbqycqqGJbWycqqGPbqAcqqG0baDcqqG5bqEcqqGGaaicqqGOaakcqqGwbGvcqqGdbWqcqqGPaqkiiaacqWF9aqpdaWcaaqaaiabd6eaonaaCaaaleqabaGaey4fIOcaaaGcbaGaemisaGeaamaalaaabaGaemyyae2aaWbaaSqabeaacqaIYaGmaaGccqWGJbWyiiGacqGF4oqCdaWgaaWcbaGaemyraueabeaaaOqaaiab+X7aTnaaBaaaleaacqWGjbqsaeqaaaaaaaa@571B@

In studying how adult mortality influences the EIR and VC it is normal to treat the ratio of mosquitoes to humans (N*/*H*) as a constant. But because the complete mosquito life cycle is explicitly modelled it is also possible (though it is not done here) to explore how adult mortality indirectly influences EIR through its effects on N*.

### Model 3, Mosquito with Plasmodium and a simplified human stage

#### Epidemiological statics

Including the dynamics of *Plasmodium *in humans allows the basic epidemiological number *R*_0 _to be calculated. This is the number of secondary infections that arise from a single initial infection in an otherwise disease-free system. The calculation is similar to that for Vectorial Capacity but now instead of allowing the introduced human to be fed on for a single day it is assumed that it is attacked throughout its infectious period of 1/*r *days (or *T*_*β *_days for eqn. 3g). Actual rather than just potentially infectious bites are now of interest, and so the efficiency of transmission from mosquitoes to humans, *b*, must also be included. Thus

R0=br VC= N∗Ha2bcθErμI,
 MathType@MTEF@5@5@+=feaafiart1ev1aaatCvAUfKttLearuWrP9MDH5MBPbIqV92AaeXatLxBI9gBaebbnrfifHhDYfgasaacH8akY=wiFfYdH8Gipec8Eeeu0xXdbba9frFj0=OqFfea0dXdd9vqai=hGuQ8kuc9pgc9s8qqaq=dirpe0xb9q8qiLsFr0=vr0=vr0dc8meaabaqaciaacaGaaeqabaqabeGadaaakeaacqqGsbGudaWgaaWcbaGaeeimaadabeaakiabg2da9maalaaabaGaemOyaigabaGaemOCaihaaiabbccaGiabbAfawjabboeadHGaaiab=1da9iabbccaGmaalaaabaGaemOta40aaWbaaSqabeaacqGHxiIkaaaakeaacqWGibasaaWaaSaaaeaacqWGHbqydaahaaWcbeqaaiabikdaYaaakiabdkgaIjabdogaJHGaciab+H7aXnaaBaaaleaacqWGfbqraeqaaaGcbaGaemOCaiNae4hVd02aaSbaaSqaaiabdMeajbqabaaaaOGaeiilaWcaaa@4882@

where N* = S* is the equilibrium number of mosquitoes when the infection is assumed to be vanishingly rare. This form of *R*_0 _(but with constant recruitment to the adult stage) was first derived by Macdonald [4].

#### Equilibrium densities

At equilibrium, mosquitoes recruit to the adult stage at a constant rate that exactly balances adult mortality (μ¯
 MathType@MTEF@5@5@+=feaafiart1ev1aaatCvAUfKttLearuWrP9MDH5MBPbIqV92AaeXatLxBI9gBaebbnrfifHhDYfgasaacH8akY=wiFfYdH8Gipec8Eeeu0xXdbba9frFj0=OqFfea0dXdd9vqai=hGuQ8kuc9pgc9s8qqaq=dirpe0xb9q8qiLsFr0=vr0=vr0dc8meaabaqaciaacaGaaeqabaqabeGadaaakeaaiiGacuWF8oqBgaqeaaaa@2E81@N*). Assume first that this is a constant unaffected by the ratio of susceptible, exposed and infectious mosquitoes; this is the normal assumption in MacDonald-type models. The equilibrium number of infectious mosquitoes (and those of other classes if required) and proportion infected humans can be calculated from eqn. 8.

x∗=R0−μSμ¯R0+acμ¯,I∗=N∗(R0−μSμ¯)R0μIμ¯θE+abrN∗HμSμ¯=(R0−μSμ¯R0)(acθEμ¯acμI+μSμI).
 MathType@MTEF@5@5@+=feaafiart1ev1aaatCvAUfKttLearuWrP9MDH5MBPbIqV92AaeXatLxBI9gBaebbnrfifHhDYfgasaacH8akY=wiFfYdH8Gipec8Eeeu0xXdbba9frFj0=OqFfea0dXdd9vqai=hGuQ8kuc9pgc9s8qqaq=dirpe0xb9q8qiLsFr0=vr0=vr0dc8meaabaqaciaacaGaaeqabaqabeGadaaakeaafaqabeGabaaabaGaemiEaG3aaWbaaSqabeaacqGHxiIkaaGccqGH9aqpdaWcaaqaaiabdkfasnaaBaaaleaacqaIWaamaeqaaOGaeyOeI0YaaSGaaeaaiiGacqWF8oqBdaWgaaWcbaGaem4uamfabeaaaOqaaiqb=X7aTzaaraaaaaqaaiabdkfasnaaBaaaleaacqaIWaamaeqaaOGaey4kaSYaaSGaaeaacqWGHbqycqWGJbWyaeaacuWF8oqBgaqeaaaaaaGaeiilaWcabaGaemysaK0aaWbaaSqabeaacqGHxiIkaaGccqGH9aqpdaWcaaqaaiabd6eaonaaCaaaleqabaGaey4fIOcaaOWaaeWaaeaacqWGsbGudaWgaaWcbaGaeGimaadabeaakiabgkHiTmaaliaabaGae8hVd02aaSbaaSqaaiabdofatbqabaaakeaacuWF8oqBgaqeaaaaaiaawIcacaGLPaaaaeaacqWGsbGudaWgaaWcbaGaeGimaadabeaakmaaliaabaGae8hVd02aaSbaaSqaaiabdMeajbqabaaakeaacuWF8oqBgaqeaiab=H7aXnaaBaaaleaacqWGfbqraeqaaaaakiabgUcaRmaaliaabaGaemyyaeMaemOyaigabaGaemOCaihaamaaliaabaGaemOta40aaWbaaSqabeaacqGHxiIkaaaakeaacqWGibasaaWaaSGaaeaacqWF8oqBdaWgaaWcbaGaem4uamfabeaaaOqaaiqb=X7aTzaaraaaaaaacqGH9aqpdaqadaqaamaalaaabaGaemOuai1aaSbaaSqaaiabicdaWaqabaGccqGHsisldaWccaqaaiab=X7aTnaaBaaaleaacqWGtbWuaeqaaaGcbaGaf8hVd0MbaebaaaaabaGaemOuai1aaSbaaSqaaiabicdaWaqabaaaaaGccaGLOaGaayzkaaWaaeWaaeaadaWcaaqaaiabdggaHjabdogaJjab=H7aXnaaBaaaleaacqWGfbqraeqaaOGaf8hVd0MbaebaaeaacqWGHbqycqWGJbWycqWF8oqBdaWgaaWcbaGaemysaKeabeaakiabgUcaRiab=X7aTnaaBaaaleaacqWGtbWuaeqaaOGae8hVd02aaSbaaSqaaiabdMeajbqabaaaaaGccaGLOaGaayzkaaGaeiOla4caaaaa@8BA2@

But if the assumption of constant N* is relaxed then changes in the parameters, and in particular the relative mortality rates experienced by different categories of adult mosquito, will affect equilibrium densities both directly through the mortality terms in eqn. 11 but also indirectly through N* and *R*_0_.

If all adult mosquito classes experience the same levels of mortality (*μ*_*I*_), and if the exposed period is so short that *θ*_*E *_is effectively one, then eqn. 11 simplifies to

x∗=R0−1R0+acμI,I∗N∗=(R0−1)R0(acμI1+acμI),
 MathType@MTEF@5@5@+=feaafiart1ev1aaatCvAUfKttLearuWrP9MDH5MBPbIqV92AaeXatLxBI9gBaebbnrfifHhDYfgasaacH8akY=wiFfYdH8Gipec8Eeeu0xXdbba9frFj0=OqFfea0dXdd9vqai=hGuQ8kuc9pgc9s8qqaq=dirpe0xb9q8qiLsFr0=vr0=vr0dc8meaabaqaciaacaGaaeqabaqabeGadaaakeaafaqabeqacaaabaGaemiEaG3aaWbaaSqabeaacqGHxiIkaaGccqGH9aqpdaWcaaqaaiabdkfasnaaBaaaleaacqaIWaamaeqaaOGaeyOeI0IaeGymaedabaGaemOuai1aaSbaaSqaaiabicdaWaqabaGccqGHRaWkdaWccaqaaiabdggaHjabdogaJbqaaGGaciab=X7aTnaaBaaaleaacqWGjbqsaeqaaaaaaaGccqqGSaalaeaadaWcaaqaaiabdMeajnaaCaaaleqabaGaey4fIOcaaaGcbaGaemOta40aaWbaaSqabeaacqGHxiIkaaaaaOGaeyypa0ZaaSaaaeaadaqadaqaaiabdkfasnaaBaaaleaacqaIWaamaeqaaOGaeyOeI0IaeGymaedacaGLOaGaayzkaaaabaGaemOuai1aaSbaaSqaaiabicdaWaqabaaaaOWaaeWaaeaadaWcaaqaamaaliaabaGaemyyaeMaem4yamgabaGae8hVd02aaSbaaSqaaiabdMeajbqabaaaaaGcbaGaeGymaeJaey4kaSYaaSGaaeaacqWGHbqycqWGJbWyaeaacqWF8oqBdaWgaaWcbaGaemysaKeabeaaaaaaaaGccaGLOaGaayzkaaGaeiilaWcaaaaa@5BC9@

which is the standard solution to the coupled MacDonald equation [5,6].

## Analysis: dynamics

The dynamics of the population models developed in this paper can be studied in two ways. First, they can be solved numerically using relatively straightforward modifications of software designed to solve systems of ordinary differential equations [29-31]. Such investigation shows the full range of equilibrium and non-equilibrium behaviour. Second they can be subject to local stability analysis which reveals the boundaries between stable and unstable regions of parameter space. However, unlike many systems of ordinary differential equations, it is seldom possible to obtain analytical stability boundaries and these have to be solved numerically. Because of the time delays in the system, non-equilibrium behaviour is usually oscillatory, and it is also possible to calculate the period of the oscillations, at least in the vicinity of the stability boundary.

The dynamics of the three models developed here is now explored, though it is again pointed out that the chief aim is to demonstrate the possible uses of this modelling approach, rather than to produce new results in mosquito-malaria population biology. Table 1 gives the basic parameter set used in numerical simulations; it is based on the biological studies referred to in the table, but should not be considered a definitive description of any particular mosquito-*Plasmodium *interaction.

**Table 1:** The parameter values used in the illustrative model runs. They were chiefly motivated by the studies listed on *Anopheles gambiae *s.s. in Africa, but do not attempt to model precisely any particular interaction.

Parameter	Symbol	Value	Unit	Source
Duration of egg stage	*T*_*O*_	1	d	[2]
Duration of larval stage	*T*_*L*_	14	d	[2]
Duration of pupal stage	*T*_*P*_	1	d	[2]
Duration of exposed stage	*T*_*E*_	10	d	[5]
Egg stage daily mortality	*μ*_*O*_	0.05	d^-1^	[43]
Larval stage daily mortality	*μ*_*L*_	0.1	d^-1^	[44]
Pupal stage daily mortality	*μ*_*P*_	0.05	d^-1^	[45]
Adult stage daily mortality	*μ*_*S*_, *μ*_*E*_, *μ*_*I*_	0.1	d^-1^	[5]
Fecundity	*λ*	30	d^-1^	[2]
Density dependent parameter	*γ*	0.001	d^-1 ^area^2^	
Transmission efficiency; mosquito to human	*b*	0.5		[5]
Transmission efficiency; human to mosquito	*c*	0.5		[5]
Human biting rate	*a*	0.3	d^-1^	[5]
Human recovery rate	*r*	0.01	d^-1^	[5]

### Model 1, Mosquito with no Plasmodium

**Figure 2:**
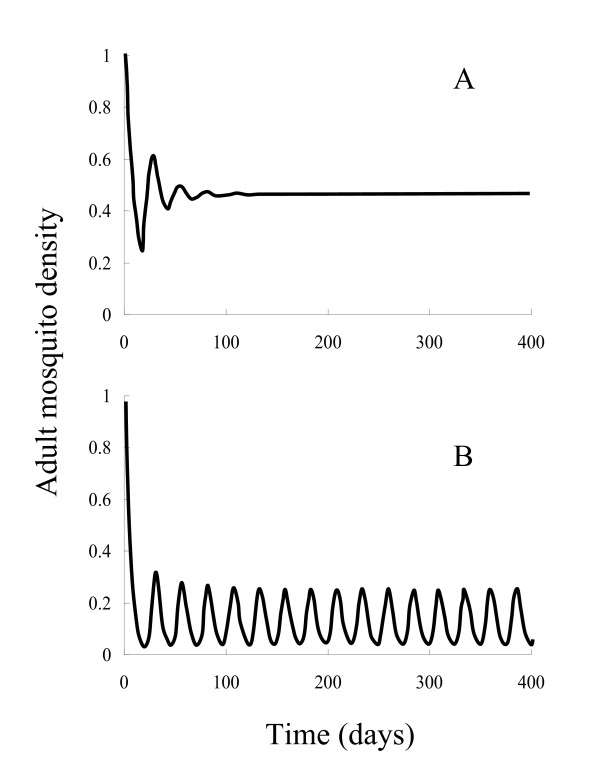
Examples of the population dynamics shown by Model 1: (A) parameters as in Table 1; (B) parameters as in Table 1 but adult fecundity (*λ*) raised to 100 and pupal period (*T*_*P*_) to 1.1.

Begin by exploring the dynamics of the simplest model without *Plasmodium*. This consists of a single-species population with overlapping generations and density-dependent mortality that acts in a delayed manner; the delay arises because there is a gap in time between density dependent mortality acting at the larval stage and any reduction in the rate at which eggs are produced which occurs only when the cohort of larvae reached maturity. Standard results from theoretical ecology [27,32] predict that a population with this type of dynamics may show a deterministically stable equilibrium, or show cyclic or more complex dynamic behaviours (possibly including chaos). Figure 2a shows that for the parameters in Table 1 a stable equilibrium is predicted, though were fecundity to be higher and developmental time delays greater than the system can show cyclic dynamics (Figure 2b).

Further insight into what determines the population dynamic behaviour can be gained from local stability analysis, a standard technique in dynamics that determines whether small perturbations from equilibrium die away or grow [27]. The presence of time lags makes the analysis a little more complicated. The Appendix shows how local stability analysis leads to an expression *f*(*η*, **P**) = 0 where *η *is a dummy variable and **P **is a vector of parameters from the population model. In general *f*(*η*, **P**) has an infinite number of roots in *η *and the system is stable in those regions of parameter space where the real parts of all roots are negative. At the stability boundary the real parts equal zero and the magnitude of any associated imaginary part is proportional to the period of the damped or divergent oscillations in the vicinity of the stability boundary.

**Figure 3:**
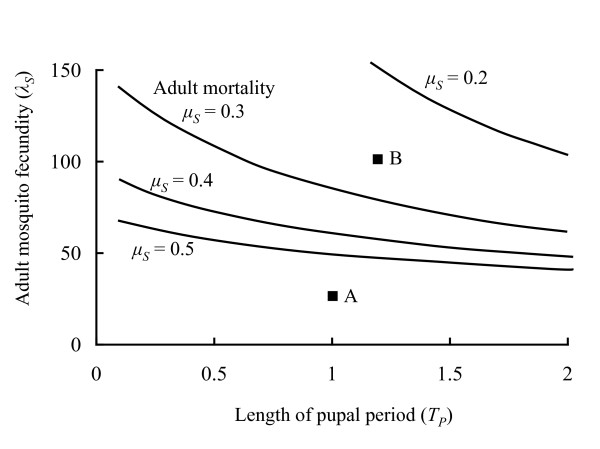
Stability analysis of Model 2: the curves (each representing different values of adult mortality as indicated on the figure) separate regions in "fecundity-pupal period space" of stable population dynamics (below the line) and cyclical population dynamics (above the line). The points A and B are the parameter values used in the two simulations with the same labels in Figure 2.

Whether the population converges on a stable equilibrium or shows population cycles is determined chiefly by three classes of parameter. Processes that tend to increase the mosquito population growth rate either by increasing fecundity (higher *λ*_*S*_) or reducing sub-adult mortality (lower *μ*_*O*_, *μ*_*L *_or *μ*_*P*_) make cyclic population dynamics more likely. Processes that reduce the time lag in the response of the population to density dependence (shorter *T*_*O*_, *T*_*L *_or *T*_*P *_for constant through stage mortality) make cycles less likely. Finally the effect of adult mortality (*μ*_*S*_) is more complex: higher mortality leads to reduced fecundity which tends to promote stability. However, a short-lived adult stage is destabilising because it can lead to a pulse or cohort of larvae that experiences high levels of mortality giving rise to a low number of adults when the cohort matures. This, in its turn, produces a relatively small cohort that experiences low levels of mortality leading to a large number of adults. A longer adult stage causes cohorts to mix and for the effects of relatively small and large cohorts to be averaged out. In general the destabilising effect of increased adult mortality is stronger than the stabilising. Figure 3 illustrates how *λ*_*S*_, *T*_*P *_and *μ*_*S *_combine together to determine the stability boundary. The strength of density dependence in this formalism (*γ*) does not influence dynamics, though this will not be true in general for arbitrary forms of density-dependence.

The effects of different mosquito control strategies can be studied by assuming they affect stage-specific density-independent mortality. Thus habitat modification or larval insecticides may increase *μ*_*O*_, *μ*_*L *_and *μ*_*P*_, while insecticidal bed nets may increase *μ*_*S*_. The consequences of different interventions or combinations of interventions on the equilibrium density of adult mosquitoes can be estimated from eqn. 6, while numerical solutions of eqn. 1 provide information on the rate at which any decrease in mosquito populations is attained.

### Model 2, Mosquito with Plasmodium

In this model the mosquito is infected with *Plasmodium *after feeding and it is assumed that a fixed proportion of humans carry the pathogen. There is thus no coupled mosquito-malaria dynamics, but *Plasmodium *does influence the mosquito population by possibly altering adult death rates (for example if exposed and infectious mosquitoes have higher mortalities, *μ*_*E*_, *μ*_*I *_> *μ*_*S*_) or reduced fecundity (*λ*_*E*_, *λ*_*I *_<*λ*_*S*_). It is unlikely that the effects of *Plasmodium *on mosquito dynamics will be large, but local stability analysis shows that where they increase average adult mortality they will tend to be destabilising, and where they decrease average fecundity they tend to be stabilising.

### Model 3, Mosquito with Plasmodium and a simplified human stage

**Figure 4:**
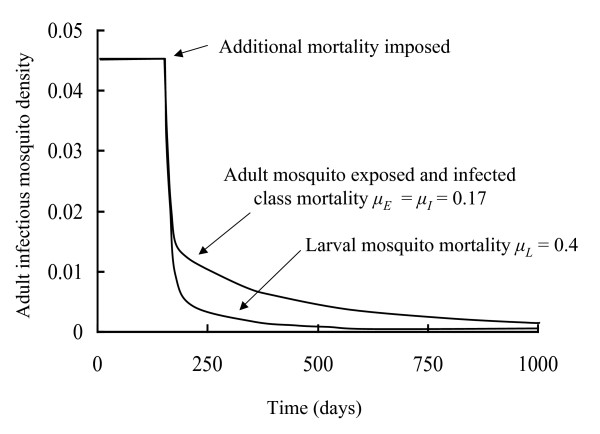
The effect of the imposition of extra mortality on the density of adult infectious mosquitoes, as predicted by Model 3. All parameters are as in Table 2 until day 200 when either larval or adult (exposed and infectious classes) mortality is increased as indicated in the figure.

**Figure 5:**
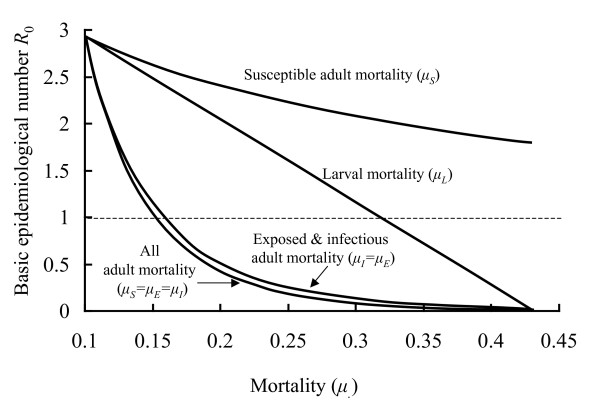
The effect of changing different mortality factors on *R*_0_, the basic epidemiological number. All parameters are as in Table 1 except for the mortality factor or factors indicated for each curve. The parasite can only persist when *R*_0 _> 1, the horizontal dashed line.

Here the fraction of infected humans is a dynamic variable coupled with the mosquito-*Plasmodium *interaction. The model can be used to explore the dynamics of disease spread in vector and human, as well as the potential effects of different control strategies. Figure 4 provides an illustration of the latter. A mosquito/*Plasmodium *interaction is assumed to be at equilibrium for the default parameters in Table 1. These parameters give rise to *R*_0 _= 2.57 and as this is greater than one the interaction is persistent. At the time indicated in the figure two different control strategies are imposed; one that increases daily larval mortality to 0.4 and the other that increases the daily mortality of exposed and infected adults to 0.17. For these parameter values *R*_0 _is no longer greater than one and the infection cannot persist. The model shows how quickly the numbers of infectious mosquitoes fall after the two different control strategies are implemented. Figure 5 summarises how imposing increasing mortality on different mosquito life history stages can influence disease persistence through *R*_0_.

Though more complex, and probably not of great applied interest, local stability analysis can also be carried out on Model 3. The results differ slightly from Model 2 because now the *Plasmodium *affects mosquito dynamics both through their direct effect on mortality and fecundity parameters (*μ*_*E*_, *μ*_*I*_, *λ*_*E*_, *λ*_*I*_) but also indirectly through changing the fraction of infected humans. An increase in adult mortality due to infection with malaria has less of a destabilising influence when the dynamics of the infection in the human population are included, because higher mortality rates of infectious mosquitoes leads to lower densities of infectious mosquitoes, which reduces the proportion of infectious humans which leads to a further decrease in the density of infectious mosquitoes.

## Discussion

Mathematical models in population biology can be broadly characterised as strategic or tactical [33]. Strategic models seek to give broad answers to general questions: in the present context to address issues such as whether vector control efforts should target the adult or juvenile mosquito life stages, or whether genetic manipulation to create a partially refractory vector could lead to the extinction of the disease. Tactical models on the other hand seek to resolve much more specific issues, for example in the current context whether *Anopheles gambiae *s.s. populations at a particular locality and time of year can be controlled by insecticide-impregnated bednets. Both approaches have strengths and weaknesses and each have distinct roles in understanding population dynamics and designing control measures. With strategic models there is a trade-off between simplicity (which normally equates with analytical tractability) and oversimplification (which implies biological unrealism).

The models developed here are strategic models, but phrased in a way that it is argued allows greater biological realism to be added for a relatively modest increase in complexity. The models can be studied analytically, though the switch to delay-differential equations makes this somewhat harder, while traditional and very well understood classical models in epidemiology and population ecology can be obtained as limiting cases. The single most useful aspect of this formalism is that the relatively fixed time delays in the system that occur as the mosquito moved through the egg, larval and pupal stage, and as the *Plasmodium *matures in the adult insect, can be entered in a transparent and natural way.

Many models of malaria-mosquito interactions do not explicitly treat the larval stages but instead assume a constant or cyclic rate of recruitment to the adult stage. One reason for this is the argument originally due to Ross [3] that interventions against the adult insect are far more efficient at reducing biting rates compared with larval interventions. This has led in recent years to a concentration of effort in treating malaria as a human disease and attacking the adult mosquito using impregnated bed-nets and by spraying in and around houses. If the primary aim of the vector component of this strategy is to prevent the *Anopheles *living long enough to transmit malaria then a detailed representation of recruitment to the adult stage is of less importance. A second justification is the ecological argument that if mosquito population size is determined by density-dependent processes acting at the larval stage, then adult recruitment will be relatively constant or show a simple relationship to meteorological drivers.

There are at least two arguments for renewed interest in larval mosquito dynamics. First, a number of authors have argued recently that the goals of programmes such as *Roll Back Malaria *can best be met by an integrated approach combining treatment of humans and interventions against both adult and larval vectors [for a review see ref [34], and references cited therein]. They point in particular to the major successes in eradicating *A. gambiae *s.l. from Brazil and Egypt in the pre-DDT era though comment that "our understanding of mosquito larval ecology has scarcely advanced since the days of Ronald Ross, leaving most of the questions that were raised over 50 years ago unanswered" [34]. Of course what is primarily required is field observations and especially experimentation, but it is hoped that the type of model developed here can assist in understanding pre-adult dynamics and improving intervention strategies.

There is much current excitement in potential new ways to control vectors by driving genes through mosquito populations that either reduce mosquito fitness, or render them incapable of transmitting malaria. This technology is still many years from implementation, and raises a variety of technical, safety and ethical issues [35]. Some of the options under consideration might involve killing or sterilising vectors at different parts of their life cycle using stage-specific promoters, or expressing genes that influence longevity or lead to only partial *Plasmodium *transmission [36]. Design, regulation or implementation of any of these strategies is likely to require an understanding of the interplay of mortality, both density-independent and density-dependent, at different stages of the lifecycle, as well as the consequences for disease dynamics. To do this models that explicitly incorporate juvenile stages are needed.

A series of simplifications have been made to obtain the models described here, some of the most important of which will be discussed. Of course simplicity *per se *is not necessarily a bad thing if it leads to greater generality and insight; the problems arise if important biological processes are omitted leading to either misleading or irrelevant results.

The basic assumption of the Lumped Age-Class approach is that demographic and life history parameters are constant within a stage. This will always be to some extent untrue, and in certain cases may lead to the omission of significant biological processes. For example, the models developed here treat all larvae as identical while it is likely that the first and last instar suffer different mortality rates. Assuming an average mortality rate across all larval instars may normally be acceptable, though in other insect systems where older larvae interfere or cannibalise younger larvae such an assumption would result in major dynamical processes being overlooked [22,37]. One option that retains the Lumped Age-Class formalism is to model larval instars separately [25], though this requires two equations for each instar.

There are at least two possible ways in which assuming constant demographic parameters within the three adult stages may be misleading. First, it has been assumed that insects feed (and in the case of susceptibles risk becoming infected) and oviposit at a constant rate throughout the adult stage. However, mosquitoes go through a gonotrophic cycle in which adults search for a blood meal, digest it, and then oviposit, before beginning the cycle anew. A typical gonotrophic cycle might last three to five days and hence an infectious mosquito will have gone through at least two or three cycles. It is highly likely that mortality rates differ over the feeding, digesting and ovipositing stages of the cycle, though while it would be nice to include these the authors are not aware of any stage-specific estimates of mortality rates (which is not surprising considering the huge challenges of estimating survival rates in the field). Moreover, some interventions such as insecticide-impregnated bed nets target mosquitoes at specific stages of the gonotrophic cycle. An extension of the models described here that explicitly represents the gonotrophic cycle is currently being developed. This is challenging because it is necessary to index infected mosquitoes by both the length of time since they acquired the infection, and by their position in the gonotrophic cycle, something that is not possible working within the basic Lumped Age-Class approach.

The assumption of constant demographic parameters in the adult stage will also be violated if mosquitoes senesce, something that is particularly important to know given the importance of longevity for disease transmission. There is some evidence for increased mortality with age in field mosquito populations [38], though again this is a difficult parameter to measure. Within the Lumped Age-Class formalism senescence can be incorporated by introducing one or more "elderly" stages, or alternatively a PDE approach could be taken.

Constant developmental periods have been assumed for both juvenile mosquitoes and for *Plasmodium *in infected adults. It is possible to relax this assumption by assuming a constant variance in the time taken to pass through a stage, or to make the length of a life history stage a dynamic variable influenced by the severity of larval competition [18]. What is harder is to allow a supplementary state variable such as fat reserves or size to be affected by larval competition and then go on to influence an adult trait such as longevity. Conceivably a reduction in population size might lead to reduced larval competition and a consequent increase in adult size and longevity. If larger insects lived longer then paradoxically reduced mosquito numbers might increase disease transmission. To address such concerns a PDE or related approach will probably be required.

As with many other strategic models of mosquito-malaria interactions a single homogeneous population has been assumed, with demographic parameters that do not vary with time. Many of the same approaches that have been used to relax these assumptions by people using other modelling strategies can also be employed here. Thus parameters can be allowed to vary seasonally, and spatial processes can be addressed by considering an array of populations on a lattice linked by dispersal, or where available specific spatial structure can be incorporated. All these changes will complicate the model, and make analytical insight and results harder to obtain. At some stage the technical complexities of solving large series of differential equations simultaneously are likely to make it better to transfer to a more traditional simulation approach, especially as the focus of the modelling shifts from strategic to tactical questions.

Finally, it is stressed that many complexities concerning the human host have been ignored. For example, it is known that biting rates vary considerably across individuals, and that this can have significant effects on dynamics [39,40]. The representation of the disease in humans as simply susceptible and infected is extremely crude. In reality people will have a variety of immunological responses that may be influenced by their genotype, and also by the genotype of pathogen they carry and whether there are infected by one or more *Plasmodium *strains [9,41,42]. These complications can be incorporated when required within this modelling framework.

## Conclusion

The Lumped Age-class formalism is a useful way of modelling mosquito-malaria interactions. Its chief advantage over other methods is that it allows the natural time lags inherent in the system to be incorporated in a straightforward and simple manner. The models are phrased as delay-differential equations which are slightly harder to work with than ordinary differential equations, though not prohibitively so. Software to solve them numerically is also widely available. Even when analytical results cannot be obtained, classical models from epidemiology and population ecology can be derived as limiting cases which assists greatly in the interpretation of numerical results. The standard static quantities in vector epidemiology, for example vectorial capacity, entomological inoculation rate, and *R*_0_, are all easily derived. The modelling framework can be expanded to incorporate more realistic *Plasmodium*-human interactions (though not pursued here). It is suggested that this approach will be particularly useful in studying the integration of control measures targeted at multiple adult and juvenile stages of the vector.

## Competing interests

The author(s) declare that they have no competing interests.

## Authors' contributions

With PAH's help, HCJG derived the models and wrote the paper; PAH performed all the numerical and stability analyses. Both authors have read and approved the final manuscript.

## Appendix: Local Stability Analysis

Model 1 is specified by two equations (eqns. 1) in the number of larval *L*(*t*) and *S*(*t*) mosquitoes. Define the equilibrium numbers of larvae and adults as L* and S* respectively and use lower case letters to denote local perturbations from the equilibrium state.

l(t)=L(t)−L∗s(t)=S(t)−S∗
 MathType@MTEF@5@5@+=feaafiart1ev1aaatCvAUfKttLearuWrP9MDH5MBPbIqV92AaeXatLxBI9gBaebbnrfifHhDYfgasaacH8akY=wiFfYdH8Gipec8Eeeu0xXdbba9frFj0=OqFfea0dXdd9vqai=hGuQ8kuc9pgc9s8qqaq=dirpe0xb9q8qiLsFr0=vr0=vr0dc8meaabaqaciaacaGaaeqabaqabeGadaaakqaabeqaaiabdYgaSjabcIcaOiabdsha0jabcMcaPiabg2da9iabdYeamjabcIcaOiabdsha0jabcMcaPiabgkHiTiabdYeamnaaCaaaleqabaGaey4fIOcaaaGcbaGaem4CamNaeiikaGIaemiDaqNaeiykaKIaeyypa0Jaem4uamLaeiikaGIaemiDaqNaeiykaKIaeyOeI0Iaem4uam1aaWbaaSqabeaacqGHxiIkaaaaaaa@46D7@

Proceeding as in Briggs et al. (1999) substitute eqn. A1 into eqn. 1 and linearise the system to obtain expressions for the change in the size of the perturbation

dl(t)dt=λSs(t−TO)θO−λSs(t−TO−TL)ω∗+λSS∗θOω∗γ∫t−TLtl(τ)dτ−μLl(t)−2γL∗l(t)ds(t)dt=λSs(t−TO−TL−TP)θOθPω∗−λSS∗θOθPω∗γ∫t−TL−TPt−TPl(τ)dτ−μAs(t)
MathType@MTEF@5@5@+=feaafiart1ev1aaatCvAUfKttLearuWrP9MDH5MBPbIqV92AaeXatLxBI9gBaebbnrfifHhDYfgasaacH8akY=wiFfYdH8Gipec8Eeeu0xXdbba9frFj0=OqFfea0dXdd9vqai=hGuQ8kuc9pgc9s8qqaq=dirpe0xb9q8qiLsFr0=vr0=vr0dc8meaabaqaciaacaGaaeqabaqabeGadaaakqaabeqaamaalaaabaGaemizaqMaemiBaWMaeiikaGIaemiDaqNaeiykaKcabaGaemizaqMaemiDaqhaaiabg2da9GGaciab=T7aSnaaBaaaleaacqWGtbWuaeqaaOGaem4CamNaeiikaGIaemiDaqNaeyOeI0Iaemivaq1aaSbaaSqaaiabd+eapbqabaGccqGGPaqkcqWF4oqCdaWgaaWcbaGaem4ta8eabeaakiabgkHiTiab=T7aSnaaBaaaleaacqWGtbWuaeqaaOGaem4CamNaeiikaGIaemiDaqNaeyOeI0Iaemivaq1aaSbaaSqaaiabd+eapbqabaGccqGHsislcqWGubavdaWgaaWcbaGaemitaWeabeaakiabcMcaPiab=L8a3naaCaaaleqabaGaey4fIOcaaOGaey4kaSIae83UdW2aaSbaaSqaaiabdofatbqabaGccqWGtbWudaahaaWcbeqaaiabgEHiQaaakiab=H7aXnaaBaaaleaacqWGpbWtaeqaaOGae8xYdC3aaWbaaSqabeaacqGHxiIkaaGccqWFZoWzdaWdXbqaaiabdYgaSjabcIcaOiab=r8a0jabcMcaPiabdsgaKjab=r8a0bWcbaGaemiDaqNaeyOeI0Iaemivaq1aaSbaaWqaaiabdYeambqabaaaleaacqWG0baDa0Gaey4kIipakiabgkHiTiab=X7aTnaaBaaaleaacqWGmbataeqaaOGaemiBaWMaeiikaGIaemiDaqNaeiykaKIaeyOeI0IaeGOmaiJae83SdCMaemitaW0aaWbaaSqabeaacqGHxiIkaaGccqWGSbaBcqGGOaakcqWG0baDcqGGPaqkaeaadaWcaaqaaiabdsgaKjabdohaZjabcIcaOiabdsha0jabcMcaPaqaaiabdsgaKjabdsha0baacqGH9aqpcqWF7oaBdaWgaaWcbaGaem4uamfabeaakiabdohaZjabcIcaOiabdsha0jabgkHiTiabdsfaunaaBaaaleaacqWGpbWtaeqaaOGaeyOeI0Iaemivaq1aaSbaaSqaaiabdYeambqabaGccqGHsislcqWGubavdaWgaaWcbaGaemiuaafabeaakiabcMcaPiab=H7aXnaaBaaaleaacqWGpbWtaeqaaOGae8hUde3aaSbaaSqaaiabdcfaqbqabaGccqWFjpWDdaahaaWcbeqaaiabgEHiQaaakiabgkHiTiab=T7aSnaaBaaaleaacqWGtbWuaeqaaOGaem4uam1aaWbaaSqabeaacqGHxiIkaaGccqWF4oqCdaWgaaWcbaGaem4ta8eabeaakiab=H7aXnaaBaaaleaacqWGqbauaeqaaOGae8xYdC3aaWbaaSqabeaacqGHxiIkaaGccqWFZoWzdaWdXbqaaiabdYgaSjabcIcaOiab=r8a0jabcMcaPiabdsgaKjab=r8a0bWcbaGaemiDaqNaeyOeI0Iaemivaq1aaSbaaWqaaiabdYeambqabaWccqGHsislcqWGubavdaWgaaadbaGaemiuaafabeaaaSqaaiabdsha0jabgkHiTiabdsfaunaaBaaameaacqWGqbauaeqaaaqdcqGHRiI8aOGaeyOeI0Iae8hVd02aaSbaaSqaaiabdgeabbqabaGccqWGZbWCcqGGOaakcqWG0baDcqGGPaqkaaaa@DE5C@

where the linear competition function given by eqn. 4 is assumed.

To determine whether the sets of perturbations increase or decay, the eigenvalues of the characteristic equation need to be determined. Because of the time lags and integrals this is done using Laplace transforms where is *η *is the dummy Laplace variable. The characteristic equation is

f(η,P)=|η−λSθOln⁡Λ(μL+ln⁡Λ)(1−e−ηTL)ΛηTL2(θO+μS/θP)+μL+2ln⁡ΛTLλSθOe−η(TO+TL)Λ−λSθOe−ηTOλSθOθPln⁡Λ(μL+ln⁡Λ)(e−η(TL+TP)−e−ηTP)ΛηTL2(θO+μS/θP)η−λSe−η(TO+TL+TP)θOθPΛ+μS|
 MathType@MTEF@5@5@+=feaafiart1ev1aaatCvAUfKttLearuWrP9MDH5MBPbIqV92AaeXatLxBI9gBamXvP5wqSXMqHnxAJn0BKvguHDwzZbqegyvzYrwyUfgarqqtubsr4rNCHbGeaGqiA8vkIkVAFgIELiFeLkFeLk=iY=Hhbbf9v8qqaqFr0xc9pk0xbba9q8WqFfeaY=biLkVcLq=JHqVepeea0=as0db9vqpepesP0xe9Fve9Fve9GapdbaqaaeGacaGaaiaabeqaamqadiabaaGcbaGaemOzayMaeiikaGccciGae83TdGMaeiilaWIaeCiuaaLaeiykaKIaeyypa0ZaaqWaaeaafaqabeGacaaabaGae83TdGMaeyOeI0Iae83UdW2aaSbaaSqaaiabdofatbqabaGccqWF4oqCdaWgaaWcbaGaem4ta8eabeaakmaalaaabaGagiiBaWMaeiOBa4Maeu4MdWKaeiikaGIae8hVd02aaSbaaSqaaiabdYeambqabaGccqGHRaWkcyGGSbaBcqGGUbGBcqqHBoatcqGGPaqkcqGGOaakcqaIXaqmcqGHsislcqWGLbqzdaahaaWcbeqaaiabgkHiTiab=D7aOjabdsfaunaaBaaameaacqWGmbataeqaaaaakiabcMcaPaqaaiabfU5amjab=D7aOjabdsfaunaaDaaaleaacqWGmbataeaacqaIYaGmaaGccqGGOaakcqWF4oqCdaWgaaWcbaGaem4ta8eabeaakiabgUcaRiab=X7aTnaaBaaaleaacqWGtbWuaeqaaOGaei4la8Iae8hUde3aaSbaaSqaaiabdcfaqbqabaGccqGGPaqkaaGaey4kaSIaeqiVd02aaSbaaSqaaiabdYeambqabaGccqGHRaWkcqaIYaGmdaWcaaqaaiGbcYgaSjabc6gaUjabfU5ambqaaiabdsfaunaaBaaaleaacqWGmbataeqaaaaaaOqaamaalaaabaGae83UdW2aaSbaaSqaaiabdofatbqabaGccqWF4oqCdaWgaaWcbaGaem4ta8eabeaakiabdwgaLnaaCaaaleqabaGaeyOeI0Iae83TdGMaeiikaGIaemivaq1aaSbaaWqaaiabd+eapbqabaWccqGHRaWkcqWGubavdaWgaaadbaGaemitaWeabeaaliabcMcaPaaaaOqaaiabfU5ambaacqGHsislcqWF7oaBdaWgaaWcbaGaem4uamfabeaakiab=H7aXnaaBaaaleaacqWGpbWtaeqaaOGaemyzau2aaWbaaSqabeaacqGHsislcqWF3oaAcqWGubavdaWgaaadbaGaem4ta8eabeaaaaaakeaacqWF7oaBdaWgaaWcbaGaem4uamfabeaakiab=H7aXnaaBaaaleaacqWGpbWtaeqaaOGae8hUde3aaSbaaSqaaiabdcfaqbqabaGcdaWcaaqaaiGbcYgaSjabc6gaUjabfU5amjabcIcaOiab=X7aTnaaBaaaleaacqWGmbataeqaaOGaey4kaSIagiiBaWMaeiOBa4Maeu4MdWKaeiykaKIaeiikaGIaemyzau2aaWbaaSqabeaacqGHsislcqWF3oaAcqGGOaakcqWGubavdaWgaaadbaGaemitaWeabeaaliabgUcaRiabdsfaunaaBaaameaacqWGqbauaeqaaSGaeiykaKcaaOGaeyOeI0Iaemyzau2aaWbaaSqabeaacqGHsislcqWF3oaAcqWGubavdaWgaaadbaGaemiuaafabeaaaaGccqGGPaqkaeaacqqHBoatcqWF3oaAcqWGubavdaqhaaWcbaGaemitaWeabaGaeGOmaidaaOGaeiikaGIae8hUde3aaSbaaSqaaiabd+eapbqabaGccqGHRaWkcqWF8oqBdaWgaaWcbaGaem4uamfabeaakiabc+caViab=H7aXnaaBaaaleaacqWGqbauaeqaaOGaeiykaKcaaaqaaiab=D7aOjabgkHiTmaalaaabaGae83UdW2aaSbaaSqaaiabdofatbqabaGccqWGLbqzdaahaaWcbeqaaiabgkHiTiab=D7aOjabcIcaOiabdsfaunaaBaaameaacqWGpbWtaeqaaSGaey4kaSIaemivaq1aaSbaaWqaaiabdYeambqabaWccqGHRaWkcqWGubavdaWgaaadbaGaemiuaafabeaaliabcMcaPaaakiab=H7aXnaaBaaaleaacqWGpbWtaeqaaOGae8hUde3aaSbaaSqaaiabdcfaqbqabaaakeaacqqHBoataaGaey4kaSIae8hVd02aaSbaaSqaaiabdofatbqabaaaaaGccaGLhWUaayjcSdaaaa@0A1C@

where the vector **P **is the set of parameters that may influence the stability boundaries (note that in this case it does not include the competition parameter *γ*).

Equivalent but more complicated expressions can be derived for Models 2 and 3.
